# ORNIPURAL^®^ as conventional therapy versus mixture of *Curcuma longa* extract and pomegranate peel extract as homeotherapy in dogs with dexamethasone-induced hepatopathy: clinicolaboratory, ultrasonographic, and histopathological monitoring

**DOI:** 10.3389/fvets.2025.1564648

**Published:** 2025-04-09

**Authors:** Arafat Khalphallah, Sabry A. Mousa, Abdulaziz H. Almuhanna, Dalia Hassan, Laila A. Al-Shuraym, Lamya Ahmed Alkeridis, Ebtsam S. Abdel-lah, Mustafa Shukry, Enas A. Abdelhafez, Enas Elmeligy

**Affiliations:** ^1^Division of Internal Medicine, Department of Animal Medicine, Faculty of Veterinary Medicine, Assiut University, Assiut, Egypt; ^2^Department of Veterinary Clinical Sciences, Faculty of Veterinary Medicine, Jordan University of Science and Technology, Irbid, Jordan; ^3^Department of Clinical Studies, College of Veterinary Medicine, King Faisal University, Al-Ahsa, Saudi Arabia; ^4^Department of Animal and Poultry Hygiene and Environmental Sanitation, Faculty of Veterinary Medicine, Assiut University, Assiut, Egypt; ^5^Department of Biology, College of Science, Princess Nourah Bint Abdulrahman University, Riyadh, Saudi Arabia; ^6^Department of Pharmacology, Faculty of Veterinary Medicine, Assiut University, Assiut, Egypt; ^7^Department of Physiology, Faculty of Veterinary Medicine, Kafrelsheikh University, Kafrelsheikh, Egypt; ^8^Department of Cell And Tissues, Faculty of Veterinary Medicine, Assiut University, Assiut, Egypt; ^9^Veterinary Teaching Hospital, Faculty of Veterinary Medicine, Assiut University, Assiut, Egypt

**Keywords:** *Curcuma longa* extract, dogs, ORNIPURAL®, liver histopathology, pomegranate peel extract, steroidal hepatopathy, ultrasonography

## Abstract

**Introduction:**

*Curcuma longa* extract and pomegranate peel extract as homeotherapy have numerous therapeutic uses, mainly for anti-inflammatory, immunomodulatory, and hepatoprotective efficacy. The current study compared ORNIPURAL^®^ (as a commercial hepatoprotective drug) and a herbal mixture of *Curcuma longa* extract and pomegranate peel extract [as homeotherapy] in dogs with dexamethasone-induced hepatopathy throughout a 42-day long-term study.

**Methods:**

The study was conducted on mongrel dogs (*n* = 30) throughout three phases of the experiment: an acclimatization phase (14 days), a steroidal-induced hepatopathy phase (14 days), and a treatment phase (14 days, i.e., either with ORNIPURAL® or with herbal mixtures). The investigated dogs undergoing complete clinical and ultrasonographic examinations as well as hematological analysis and serum hepatorenal biomarkers that were estimated in days 0 (control group), 7 (hepatopathy group), 14 (hepatopathy group), 21 (treatment group), and 28 (treatment group). Histopathology of the liver was conducted for some dogs on days 0, 14, and 28 after the euthanization of these animals.

**Results and conclusion:**

The present study reported the most remarkable efficacy of both ORNIPURAL^®^ and a herbal mixture of *Curcuma longa* extract and pomegranate peel extract as hepatoprotective medicaments in the therapy of dexamethasone-induced fatty liver in dogs. Therefore, a 14-day treatment with either a herbal mixture or ORNIPURAL^®^ in treated dogs (treatment groups) induced an unmistakable improvement in their clinical status, blood pictures, and serum hepatorenal parameters as well as characteristic sonographic and histopathological findings compared with those in dexamethasone-induced hepatic lipidosis (hepatopathy groups). Compared to dogs treated with ORNIPURAL^®^, this clinical improvement was more evident in dogs treated with an herbal mixture. Moreover, no significant alterations in blood pictures and serum hepatorenal indices were demonstrated between ORNIPURAL^®^ and herbal-treated dogs. Overall, the herbal mix of *Curcuma longa* extract and pomegranate peel extract had higher efficacy and greater potency than conventional therapy that uses ORNIPURAL® in treating dogs with hepatopathy. The study also recommended the parallel use of this herbal mixture as well as ORNIPURAL® in long-term therapeutic strategies in dogs with dexamethasone-induced fatty liver as both minimized dexamethasone side effects. Ultrasonography alone was not enough to evaluate hepatobiliary disorders in canines.

## 1 Introduction

Hepatobiliary diseases are commonly reported in dogs, and they are classified into four types: parenchymal, vascular, biliary, and neoplastic (1, 2). Liver diseases persist for an extended period in dogs. Moreover, these subclinical animals usually undergo regular biochemical examinations; thus, they are challenging to diagnose using current screening methods (3).

Specific clinical signs are only recognized at an advanced stage, where the hepatocyte damage becomes severe, and treatment at this stage is less effective (1, 4).

Primary hepatitis is the most severe liver disease in dogs. Acute and chronic hepatitis are the most common forms (5). Acute hepatitis can be produced by the administration of various drugs, ingestion of toxins, and through infectious agents like leptospira, canine adenovirus-1 virus, *Clostridium piliformis*, a septic bacterial disease, canine herps virus, and *Toxoplasma gondii* (6). Cholelithiasis in dogs is uncommon. This disease is frequently caused by an ascending infection by bacteria from the colon through the common bile duct or by a hematogenous route (7, 8). Bile stasis and cholelithiasis are risk factors for cholecystitis (9).

Genetic predisposition and patient history (breed, age, and sex) are used to diagnose canine hepatobiliary disorders. Female dogs are more likely than male dogs to suffer from acute and chronic hepatitis, which usually affects middle-aged to older canines. Dogs older than 5 years old are more likely to develop chronic cirrhosis and hepatitis (4). The age of dogs diagnosed with lymphoma ranged from 6 to 9 years, with some variation according to type of lymphoma and breed (10).

A well-established diagnostic procedure for evaluating the liver size, shape, and echogenicity in dogs with liver disease, abdominal ultrasonography is regarded as one of the most accurate and non-invasive diagnostic techniques available (4, 11, 12).

Several hepatic diseases that affect dogs and cats and result in localized, multifocal, or diffuse parenchymal changes have been characterized by ultrasound characteristics of hepatic disorders in small animal practice. Hepatic size and shape, ultrasound-beam attenuation, parenchymal echogenicity, and the distribution of various hepatic anomalies were often included in the examination of the liver. Majority of the alterations do not indicate a specific process, even if several of these illnesses exhibited distinctive ultrasonographic markers. Clinical presentation, blood test results, ultrasonographic findings, and cytological or histopathologic data are typically combined to provide a more definitive diagnosis (13, 14).

Liver ultrasonographic characteristics showed mild to moderate alterations in, respectively, 30 and 40% of dogs with chronic hepatitis. Several studies have shown that routine clinical ultrasound is insufficient for detecting specific canine liver diseases, but ultrasonography is still the most commonly used tool for evaluating an animal’s liver. When considering a liver biopsy for histology, dogs with normal to mild ultrasonography liver abnormalities and persistently increased liver enzymes should exercise caution to prevent a misinterpretation of chronic hepatitis (4, 15).

In dogs with allergic, anaphylactic, autoimmune, and other conditions, corticosteroid or glucocorticoid medication has been used extensively since 1948. Cortisone, a synthetic version of the hormone produced by the adrenal glands, is the offender. Unfortunately, several issues about the usage of corticosteroids are documented in the 2 years that followed 1948. Steroid hepatopathy, or changes in the morphology of the liver, is a prevalent pathology in dogs receiving corticosteroid therapy (4, 16).

Nevertheless, it has been shown that overusing glucocorticoids can result in various adverse consequences in diverse animals (17). Even at moderate doses, long-term use of glucocorticoids may cause hyperadrenocorticism, a sign of Cushing’s illness (18, 19). Dogs with steroid hepatopathy have a unique condition marked by changes in the structure and function of their livers. In dogs, it often appeared two to 3 days after corticosteroid administration (4, 16).

Dogs are susceptible to developing glucocorticoid-induced hepatopathy (16, 20), which manifests biologically as elevated serum activities of alanine aminotransferase (ALT) and alkaline phosphatase (ALP) (4, 21), histopathologically as cytoplasmic vacuolation (22), and clinically as lethargy, polyphagia, polydipsia, polyuria, generalized muscle wasting, semisolid feces, rough hair, and skin rash (4).

Various phytomedicines and polyherbal formulations are used to prevent and treat hepatotoxicity and other liver disorders, so 170 hepatogenic and hepatoprotective medicinal plants are reviewed from multiple sources. Several phytochemicals found in medicinal plants have strong antioxidant properties (23–25).

Turmeric, a powder derived from the *Curcuma longa* plant, had been used for centuries as an aspic to impart a distinct flavor and yellow color to curry. *C. longa*-dried rhizomes are high in polyphenolic compounds. *C. longa* toxicity is discovered to be very low (26). Ayurvedic medicine uses turmeric because of its anti-inflammatory qualities (27, 28). Recently, it has been used as an anti-diabetic, antibacterial, anti-inflammatory, antioxidant, anti-cancer, anti-allergic, antiprotozoal, and wound-healing agent (28, 29). Salama et al. (30) stated that the ethanol extract of *C. longa* rhizomes is being investigated as a potential therapy for liver cirrhosis. Tumeric is a rhizomatous herbaceous perennial plant used as a significant part of Ayurvedic medicine. It has anti-inflammatory qualities and to cure some internal illnesses, including liver disorders, common colds, throat infections, and digestive issues (31). Turmeric dry rhizome has been reported to have anti-inflammatory and antioxidant properties, regulating bile functions, anticarcinogenic properties, and anti-hepatotoxic activity. It could also be a functional food because it protects cells from damage. Curcumin, demetoxicurcumin, and bisdemetoxicurcumin are all curcuminoid pigments found in turmeric. Turmeric inhibited lipid peroxidation, a degenerative process involving free radicals and polyunsaturated fatty acids, mainly arachidonic. The peroxidative effect on polyunsaturated fatty acids caused hepatic pathogenesis; Turmeric has been shown to benefit body weight (28, 32). Because of its strong antioxidative and anti-inflammatory qualities, *C. longa* has been used in animal experiments to treat various diseases, including cancer, autoimmunity, cardiac disease, neurological disease, gallstones, and others (28, 33, 34).

*Punica granatum* L., commonly called pomegranate, was recently described as nature’s powder fruit. Pomegranate peel has hepatoprotective properties and significantly lowered alkaline phosphate and bilirubin levels in diseased animals (35). The plant has medicinal properties such as anti-aging, anti-cancer, anti-diabetic, cardio-protective, lipid-lowering, gastroprotective, and hepatoprotective (36, 37). Pomegranate is widely used in pharmaceutical formulations, cosmetics, and food seasonings. Pomegranate is a healing food with numerous health benefits dating back to ancient times (38). Pomegranate is an essential supplier of polyphenols with potent antioxidant properties, including phenolic acid, ellagic acid, tannins, flavonoids, and anthocyanins (39). Pomegranate is an anthelmintic, antidiarrheal agent, antioxidant activity, and has a practical effect against liver fibrosis (40).

ORNIPURAL^®^, as a well-established product for the treatment of hepatic disorders in pet animals, is used to control fatty liver in layers and transient dairy cows or to support and revitalize the liver and kidney in poultry subjected to high mycotoxin levels in their feed (41, 42). On the other hand, hepatic disorders in the pet sector are commonly treated with conventional therapeutics such as ORNIPURAL^®^ injections. The product is widely used to treat liver disorders of both infectious and non-infectious causes (43). Accordingly, the current study compared ORNIPURAL^®^ (as a commercial and conventional hepatoprotective drug) and a herbal mixture of *C. longa* extract and pomegranate peel extract [as homeotherapy] in dogs with dexamethasone-induced hepatopathy throughout a 42-day long-term study throughout three phases of experiment: an acclimatization phase (14 days), a steroidal-induced hepatopathy phase (14 days), and treatment phase (14 days; either with ORNIPURAL^®^ or with herbal mixtures).

## 2 Materials

### 2.1 Animals

The study was conducted on 30 mongrel dogs (*n* = 30) weighing 12–25 kg and their ages between 1 and 4 years. These animals were observed for 2 weeks before the start of the experiment (acclimatization phase).

### 2.2 Experimental protocol and therapeutic strategy

The therapeutic strategy was divided into three phases: acclimatization phase, steroidal-induced hepatopathy phase, and treatment phase, that is, either with an herbal mixture or with ORNIPURAL^®^ (Supplementary Table S1).

#### 2.2.1 Acclimatization phase (14 days)

Each dog was housed in a single kennel with natural light and temperature during this phase. All dogs have been treated with Biocan R^®^ as rabies vaccination (1 mL/dog S/C, inactivated vaccine, Bioveta, a. s., Czech Republic). Deworming of the investigated animals with Drontal^®^ plus (one tablet/10 kg per os; each tablet for large dogs contains 136.0 mg praziquantel, 136.0 mg pyrantel base as pyrantel pamoate, and 680.4 mg febantel, Bayer AG, 51368 Leverkusen, Germany) and DECTOMAX^®^ (doramectin 10 mg/ml, 1 ml/50 kg S/C, Zoetis, USA) (1 ml/50 kg). These treatments have been repeated for each dog twice, that is, on days 1 and 14. Food was provided twice a day through vegetables, that is, potatoes, carrots, and zucchini, and chicken cooked with bread or rice. Water was available as ad libitum in a metal container during the day. All animals were clinically examined during this phase to assess their physiological status by estimating their temperature, pulse, respiration, and mucous membranes. All animals were individually housed during the acclimatization phase for 14 days before the steroidal-induced hepatopathy phase began. On the 14th day of the acclimatization phase, the investigated dogs (*n* = 30) were clinically and ultrasonographically examined; then whole blood and serum samples were collected as well. Out of the 30 dogs, euthanization of one was done later for hepatic histopathology. Euthanization was conducted using an overdose of anesthesia by both ketamine 8 ml/20 kg BWt (ketamine 10%, Alfasan, Indian, Egypt) and xylazine 4 ml/20 kg BWt (Xylaject 2%, ADWIA Co., Cairo, Egypt) in a single IV dose These animals at the 14th day of the acclimatization phase were considered the control group (Cont^gr^; *n* = 30; prior to euthanization) of this study and was referred as day zero (day 0).

#### 2.2.2 Steroidal-induced hepatopathy phase (dexamethasone-induced hepatopathy phase) (14 days)

Afterward, experimental induction of hepatopathy was achieved by intramuscular (i.m.) injection of all dogs with dexamethasone 5 (dexamethasone sodium phosphate 5 mg/ml, Vétoquinol, Québec, Canada) at a dose of 1 mg/dog/day for successive 14 days according to Sobczak-Filipiak et al. (44) starting for day 1 until day 14 where they named steroidal-induced hepatopathy group (DexaHepato^gr^; *n* = 29). Dogs were clinically and ultrasonographically examined throughout this period, and whole blood and serum samples were collected on days 7 and 14 for hematological and biochemical assays. Thereafter, one animal from each group was euthanized on the 14th day for hepatic histopathology, according to Suvarna et al. (45). As mentioned above, euthanization was conducted using an overdose of anesthesia by both ketamine and xylazine.

#### 2.2.3 Treatment phase (14 days)

At the end of 14 days of steroidal-induced hepatopathy phase, the investigated dogs [DexaHepato^gr^; *n* = 28] were divided into two equal groups (*n* = 14 for each), whereas each dog in these groups received treatments for 14 days (continuous or every other day) either with a herbal mixture of pomegranate peel extract and *C. longa* extract and thus they named herbal mixture-treated group [Herb^mix-gr^; *n* = 14] or with ORNIPURAL^®^ and thus they named ORNIPURAL^®^-treated group [every other day, that is, 7-day treatment; Ornip^gr^; *n* = 14].

Regarding the herbal mixture-treated group (Herb^mix-gr^), *C. longa* rhizomes and pomegranate peel were obtained from an herbal plant store called (Haraz) and prepared according to Salama et al. (30) and Reda et al. (46), respectively, and the dose converted from rat to dog, according to Paget and Barnes (47). Pomegranate peel extract and *C. longa* extract were added to the control diet for 14 days at daily doses of 0.06 gm/kg and 0.15 gm/kg, respectively. Pomegranate peel extract (2 gm/1 kg /day) and *C. longa* extract (15 g/1 kg of food/day) were added to the control diet for 14 successive days, starting from day 15 up to day 28. On the other side in ORNIPURAL^®^-treated group (Ornip^gr^), ORNIPURAL^®^ (Vétoquinol SA Laboratory, Paris) as a patent hepatoprotection therapeutic drug, was composed of betaine, arginine (hydrochloride), ornithine (hydrochloride), citrulline, sorbitol (E420), and meta cresol. It was used as an i.m. injection dose of 3 ml/dog/48 h, where the dose was repeated five times for 2 days. Throughout this phase, all dogs, either from Herb^mix-gr^ (*n* = 14) or Ornip^gr^ (*n* = 14), were clinically and ultrasonographically examined, and whole blood and serum samples were collected on days 21 and 28 for hematological and biochemical assays. Thereafter, one animal from each group was euthanized on day 28 for hepatic histopathology. As mentioned above, euthanization was conducted using an overdose of anesthesia by both ketamine and xylazine.

### 2.3 Samples

Blood samples were collected from each dog’s cephalic vein using a hypodermic needle and separated into two parts; whole blood samples were collected on ethylenediamine tetra-acetic acid and stored at 4°C until analysis, and blood serum samples were collected on plain vacutainer tubes and stored at −20°C until analysis. All precautions for collecting and preparing samples to achieve an accurate assessment of hematological and biochemical indices were considered as described before (48).

### 2.4 Clinical examination

According to Sturgess, all experimental dogs were exposed to a thorough clinical examination including temperature, pulse rate, respiratory rate, and mucous membranes (49).

### 2.5 Whole blood picture indices

Various hematological indices, including red blood corpuscles (RBCs), hemoglobin (Hb), packed cell volume (PCV), and total leucocytic count (TLC), were measured by a fully automated blood cell counter machine (Medonic CA620 Vet Hematology Analyzer, Stockholm, Sweden). Differential leucocytic count (DLC) was done according to the method described by Schalm and Jain (50) and Teske (51).

### 2.6 Liver functions indices

The Spectro Ultraviolet-Vis RS spectrophotometer (Labomed, Inc., Los Angeles, CA, USA) was used to determine blood concentrations of total protein (TP), albumin, liver enzymes, that is, aspartate aminotransferase (AST), ALT and ALP, total bilirubin, and direct bilirubin. Serum globulin was determined by the subtraction of albumin from TP. All kits and reagents were obtained from BioMed commercial kits (Cairo, Egypt) for TP and albumins, Gamma Trade Company (Cairo, Egypt) for AST, total bilirubin and direct bilirubin, and Spectrum commercial kits (Cairo, Egypt) for ALT and ALP.

### 2.7 Kidney function indicators

The kidney function indicators, including blood urea nitrogen (BUN) and creatinine (Cr), were estimated using Bioscience commercial kits (Cairo, Egypt) and BioMed commercial kits (Cairo, Egypt), respectively, by spectrophotometric assays (Spectro Ultraviolet-Vis RS spectrophotometer; Labomed, Inc., Los Angeles, CA, USA).

### 2.8 Ultrasonographic examination

The ultrasonographic examination was performed on sedated animals after the application of transmission gel according to Penninck and d’Anjou (14), Mannion (52), Rademacher (53), and Ahmed et al. (54) using a 6–10 MHz convex transducer, where the frequency was changed according to the examination’s requirement, connected with ultrasound apparatus with multifrequency for the larger or older one (MyLab™One VET, Esaote, Italy). Food should be withheld before the examination (52, 53).

### 2.9 Histopathological examination

According to Suvarna et al. (45), dogs were euthanized on days 0 (*n* = 1), 14 (*n* = 1), and 28 (*n* = 2) for histopathological examination. Liver samples were removed and sent for cytology and histopathology in a sterile, closed container with 10% buffered formalized saline (45, 55).

Stain sections were inspected for any pathological alterations in the tissues, including necrosis, apoptosis, inflammation, and circulatory disruptions. For each studied organ, the description of histomorphological alterations in five fields per section was used to calculate the histopathological lesion grading according to Gibson-Corley et al. (56).

### 2.10 Statistical analysis

Data were analyzed using SPSS statistical software program for Windows version 10.0.1 (SPSS Inc., Chicago, IL., USA). The normal distribution of all parameters was tested using Kolmogorov–Smirnov normality test. All parameters were normally distributed. The obtained data were described as mean ± standard deviation (SD). A general linear model repeated measures analysis of variance (ANOVA) was used to analyze the data from the clinical examination and laboratory analyses, and the significance level of results was set at *p* < 0.05. The significance of differences was assessed between the means in selected sampling days, that is, days 0 [Cont^gr^], 7 [DexaHepato^gr^], 14 [DexaHepato^gr^], 21 [Herb^mix-gr^ or Ornip^gr^], and 28 [Herb^mix-gr^ or Ornip^gr^].

## 3 Results

### 3.1 Clinical findings

**Figure 1:**
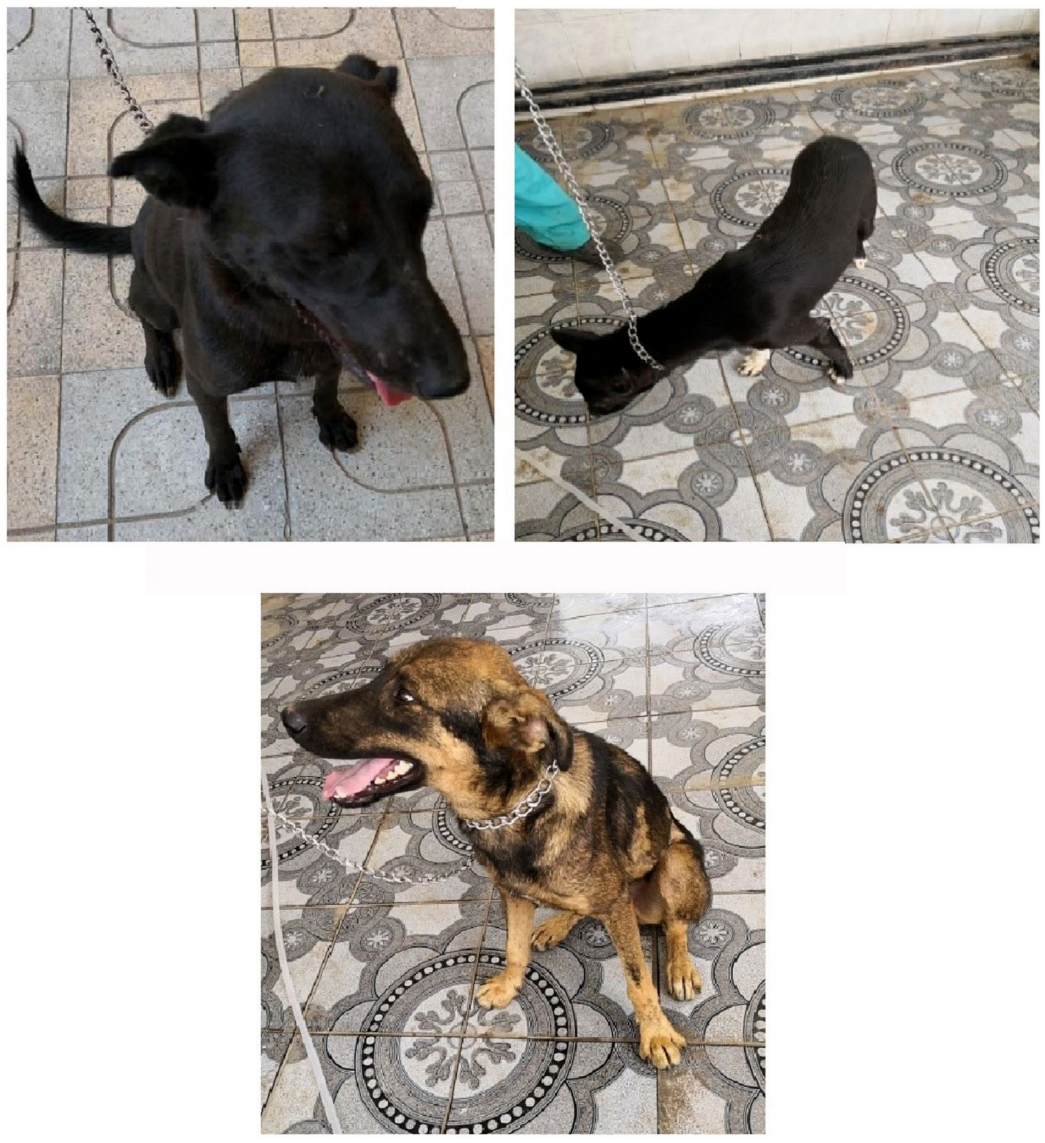
The image shows heathy control dogs in the control group (day 0). They were active and alert with healthy skin, no signs of dehydration, and suitable body weights.

**Figure 2:**
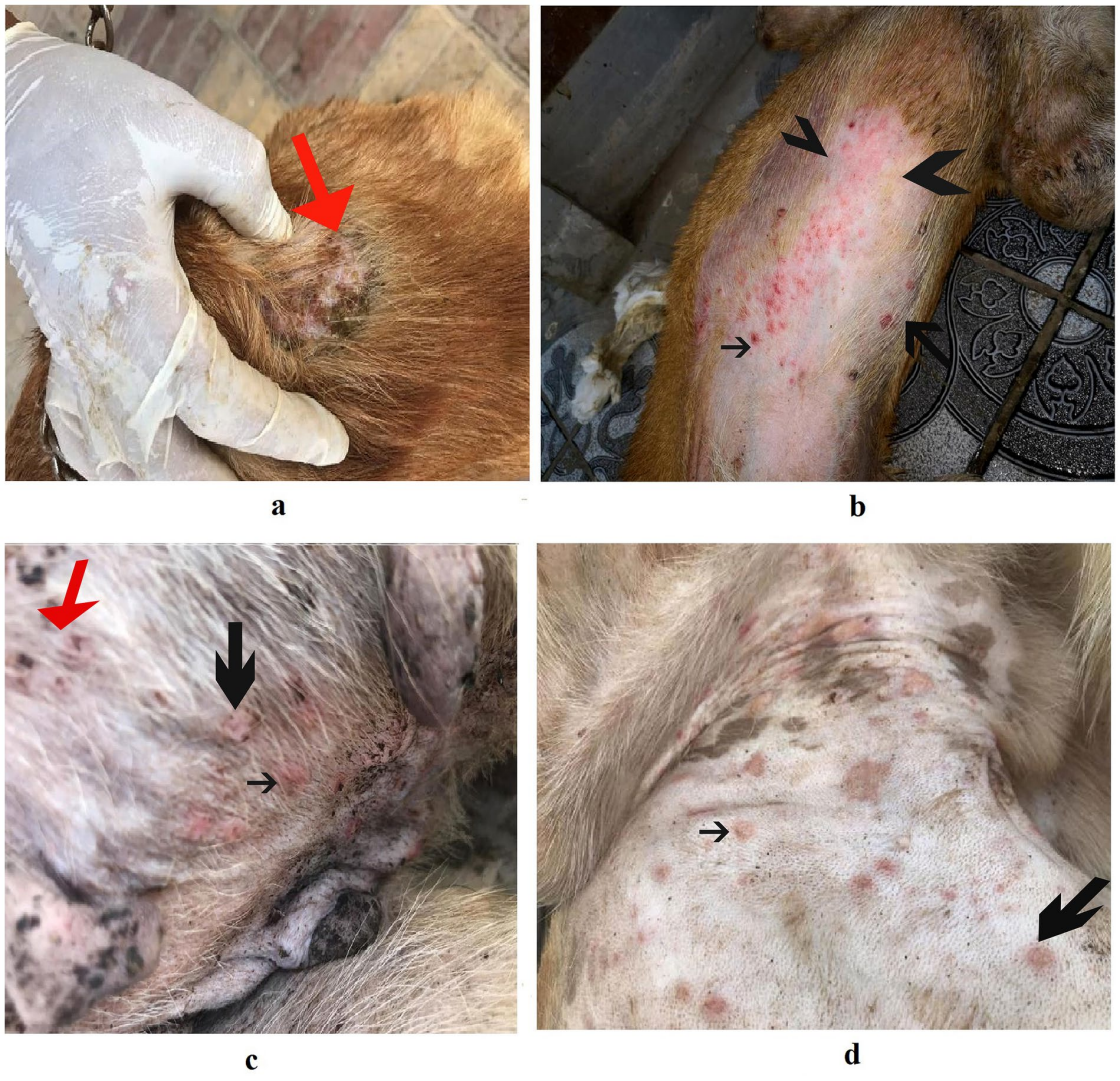
The image shows dogs in the steroidal-induced hepatopathy group after 7 days following dexamethasone injection (DexaHepato^gr^; day 7). The hepatopathy-affected dogs showed the development of skin abscesses (black arrows), skin rashes (blackhead arrows), and alopecia (red arrows).

**Figure 3:**
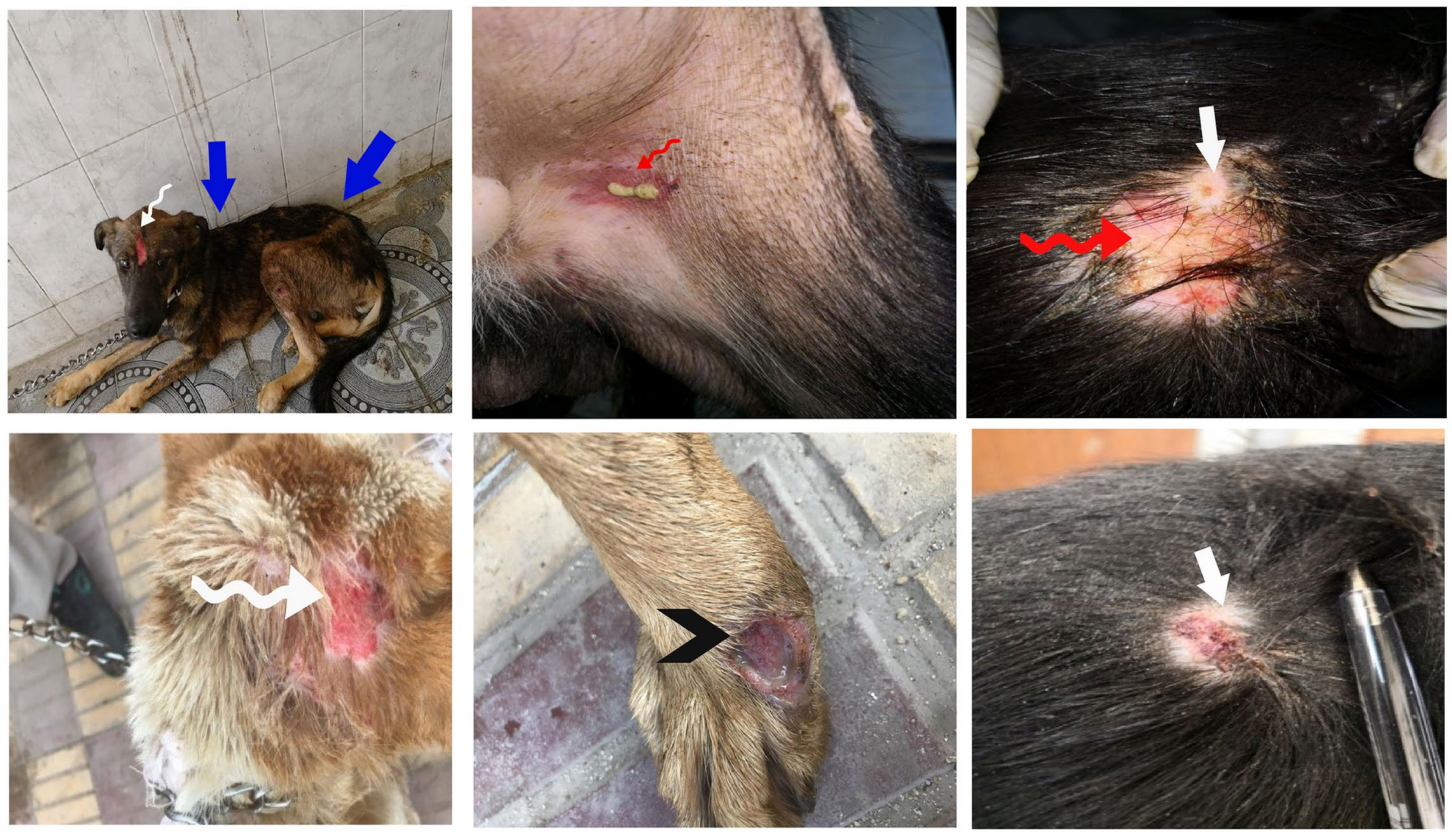
The image shows dogs in the steroidal-induced hepatopathy group after 14 days following dexamethasone injection (DexaHepato^gr^; day 14). The severity of these clinical findings was more evident on day 14 than on day 7 in DexaHepato^gr^. The hepatopathy-affected dogs showed skin lacerations (red curved arrows), ulcers (white curved arrows), and ruptured dermal abscesses (blackhead arrows). Lethargy and loss of body conditions (blue arrows) and areas of alopecia (white arrows) were observed more than on day 14 of DexaHepato^gr^.

Dogs in the control group (Day 0; Cont^gr^), either in the herbal mixture or the ORNIPURAL® group, showed normal appetite with improved feed and water intake (Figure 1). Their activities and normal gait were observable. No other abnormalities were reported, including emaciation, stunted growth, skin rashes, skin alopecia, cutaneous abscessation, ruptured dermal abscesses, skin lacerations, lethargy, and/or dehydration (Supplementary Table S2). After dexamethasone injection (DexaHepato^gr^), the dogs showed dramatic changes in their clinical findings (Figures 2, 3) either in an herbal group or ORNIPURAL® group, whereas significant (*p* < 0.05) reduction in their body weights and their appetite scores at days 7 and 14 when compared with their values at day 0 (Cont^gr^) (Table 1). These dramatic changes included lethargy, the development of skin abscesses and skin rashes, alopecia, and dehydration. The severity of these clinical findings was more evident on day 14 (Figure 3) than on day 7 (Figure 2) in DexaHepato^gr^. Skin lacerations or ulcers and ruptured dermal abscesses were seen in DexaHepato^gr^ on day 14 (Figure 3; Supplementary Table S2).

**Table 1:** Mean values (M ± SD) of body weight, appetite score, temperature, pulse, and respiration in investigated dogs.

	Body weight (kg)					Appetite score					Temperature (°C) (38–39) (49)					Pulse (Beat/min) (90–100) (57)					Respiration (/min) (15–30) (57)				
	Day 0^#^	Day 7^*^	Day 14^*^	Day 21^¶^	Day 28^¶^	Day 0^#^	Day 7^*^	Day 14^*^	Day 21^¶^	Day 28^¶^	Day 0^#^	Day 7^*^	Day 14^*^	Day 21^¶^	Day 28^¶^	Day 0^#^	Day 7^*^	Day 14^*^	Day 21^¶^	Day 28^¶^	Day 0^#^	Day 7^*^	Day 14^*^	Day 21^¶^	Day 28^¶^
Cont^gr^ (*n* = 30)	25.23 ± 2.16^a^					2.96 ± 0.13^a^					38.66 ± 0.68^a^					92.66 ± 2.5^a^					26.02 ± 3.84^a^				
DexaHepato^gr^ (*n* = 29)		18.44 ± 2.87^b^	11.03 ± 2.11^c^				1.81 ± 0.43^c^	1.09 ± 0.62^d^				38.62 ± 0.60^a^	38.70 ± 0.63^a^				90.66 ± 1.88^a^	91.52 ± 2.13^a^				25.14 ± 3.54^a^	26.34 ± 2.86^a^		
Herb^mix-gr^ (*n* = 14)				19.88 ± 1.66^b^	27.72 ± 1.66^a^				2.50 ± 0.13^b^	2.92 ± 0.13^a^				38.74 ± 0.60^a^	38.71 ± 0.66^a^				91.42 ± 2.05^a^	91.86 ± 1.91^a^				24.04 ± 2.61^a^	24.71 ± 2.81^a^
Ornip^gr^ (*n* = 14)				12.92 ± 2.15^c^	15.72 ± 1.66^b^				2.03 ± 0.21^c^	2.52 ± 0.16^b^				38.68 ± 0.52^a^	38.70 ± 0.51^a^				89.02 ± 2.66^a^	89.46 ± 2.44^a^				25.11 ± 3.06^a^	25.22 ± 2.91^a^

Cont^gr^: Control group. DexaHepato^gr^: steroid-induced hepatopathy group. Herb^mix-gr^: Herbal mixture-treated group. Ornip^gr^: ORNIPURAL® treated group. ^#^Control. ^*^Dexamethasone treatment. ^¶^Herbal mixture or ORNIPURAL® treatment. ^a-d^Means with different superscript letters in different sampling times were significantly different (*p* < 0.05) between Cont^gr^, D_exa_Hepato^gr^, Herb^mix-gr^, and Ornip^gr^ in days 0, 7, 14, 21, and 28—reference values according to Sturgess (49); Sastry (57).

**Figure 4:**
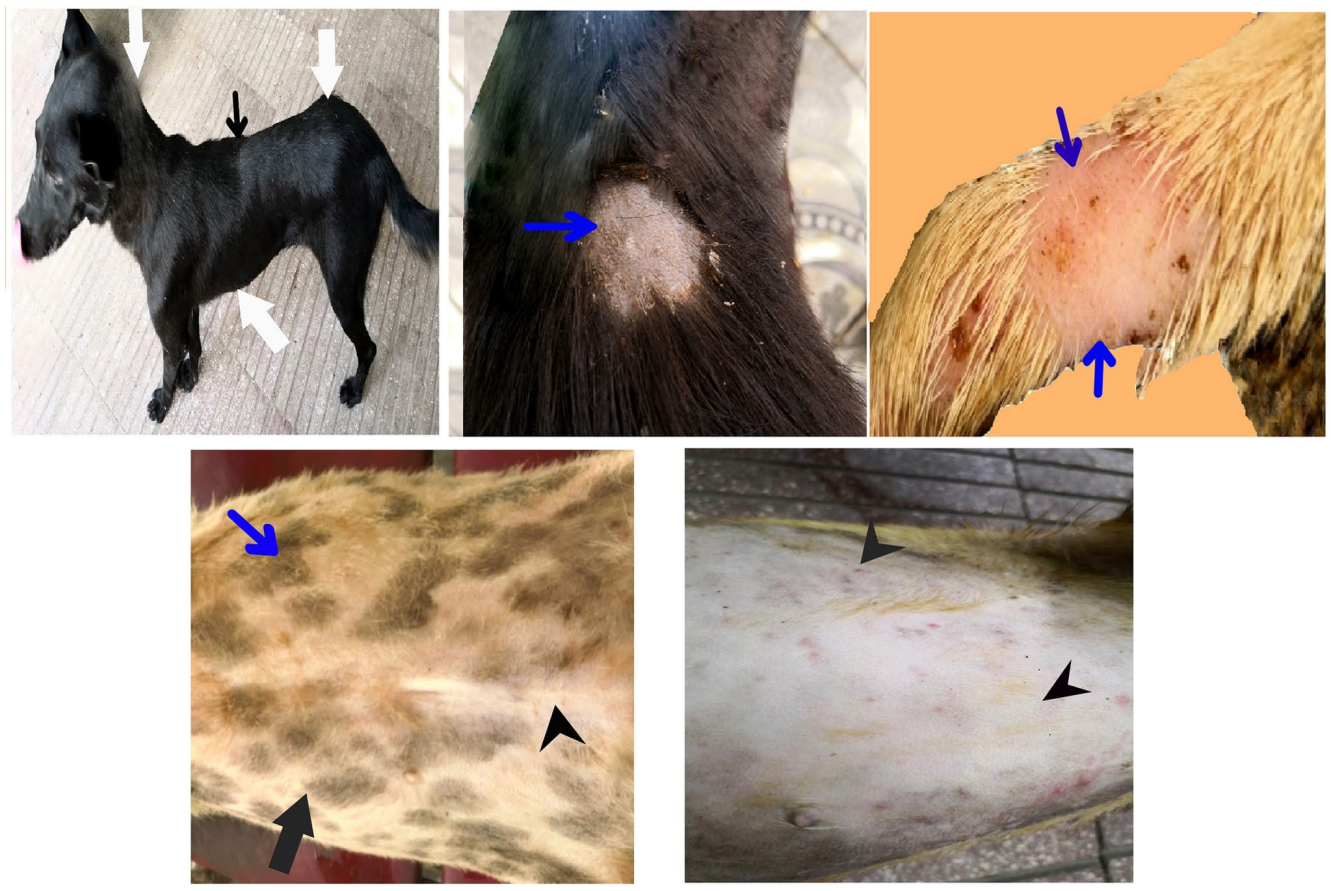
The image shows dogs in the herbal mixture-treated group (Herb^mix-gr^) after 7 days of treatment with an herbal mixture of pomegranate peel extract and *C. longa* extract (Herb^mix-gr^; day 21). The affected dogs expressed clear improvement in their body conditions (white arrows) and no lethargy with almost complete disappearance of alopecia (black arrows), skin rashes, skin lacerations (blue arrows), and dehydration signs as well as complete healing of ruptured skin abscess (black head arrows) were observed.

**Figure 5:**
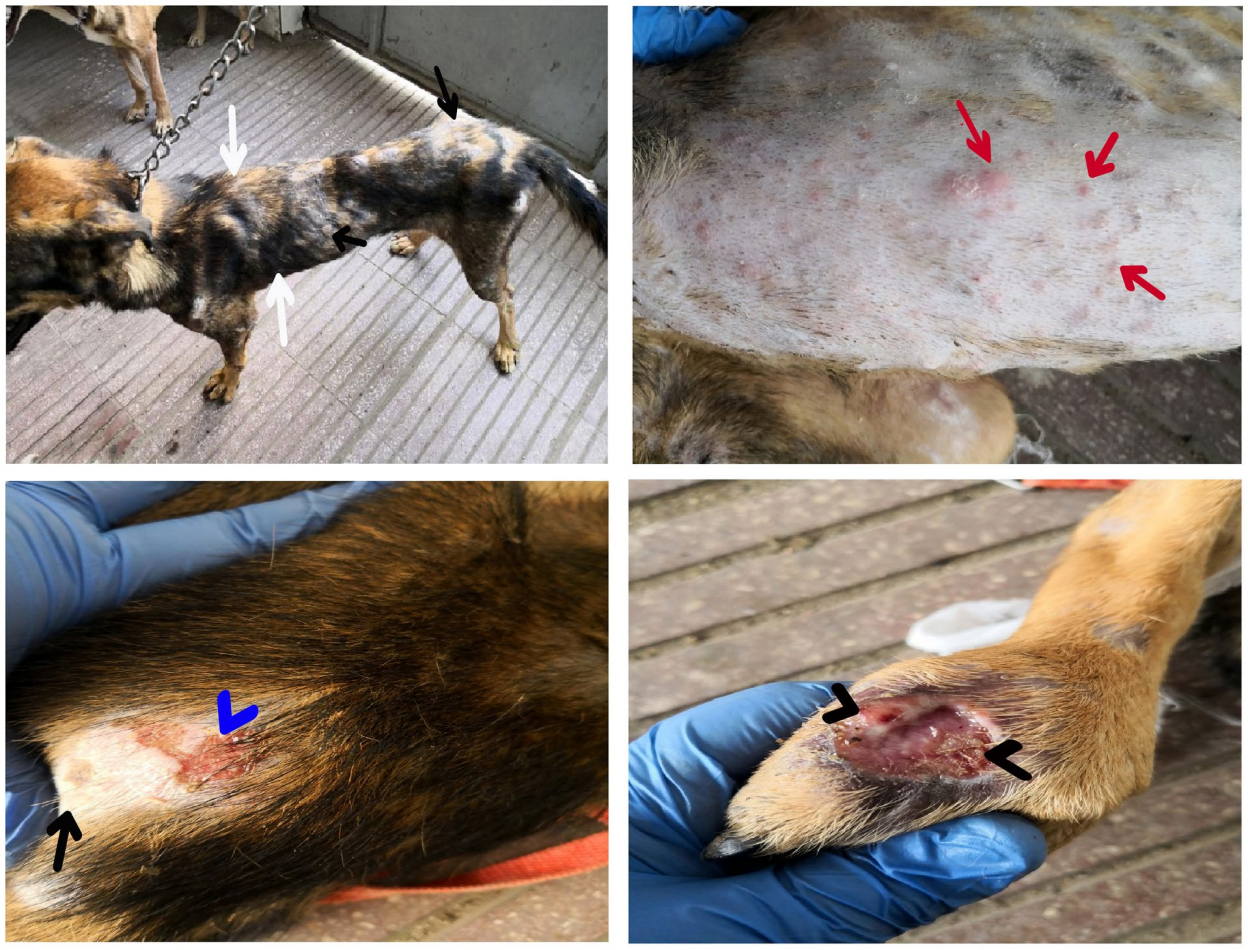
The image shows dogs in ORNIPURAL® treated group (Ornip^gr^) after 7 days following ORNIPURAL® (Ornip^gr^; day 21) for five-time treatment/10 days with ORNIPURAL® at one-day intervals. The affected dogs were still suffering from poor health conditions, emaciation, and loss of body weight (white arrows). Areas of alopecia (black arrows), skin rashes (blue arrows), skin lacerations (blue head arrows), and ruptured skin abscesses (black arrows), as well as skin abscesses (red arrows), still appeared.

**Figure 6:**
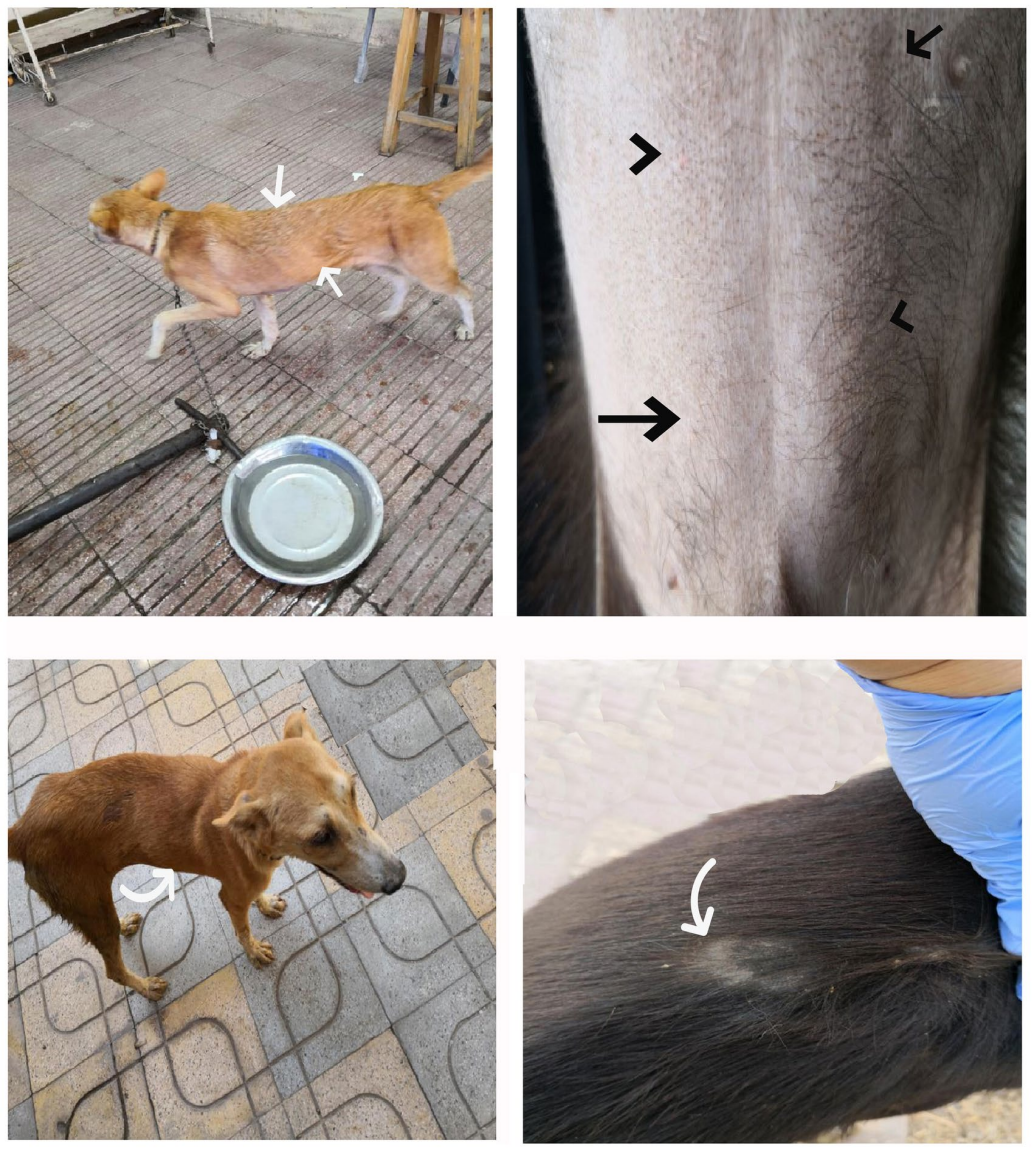
The image shows dogs in the herbal mixture-treated group (Herb^mix-gr^) after 14 days following treatment with a herbal mixture of pomegranate peel extract and *C. longa* extract (Herb^mix-gr^; day 28). The examined dogs on day 28, after treatment with the herbal mixture (Herb^mix-gr^) for 14 days, restored their body conditions and body weights (white arrows) with the healthy appearance of their skin. Complete healing of skin lacerations (black head arrows) and disappearance of skin abscesses (black arrows) and alopecia (curved white arrows) were observed.

**Figure 7:**
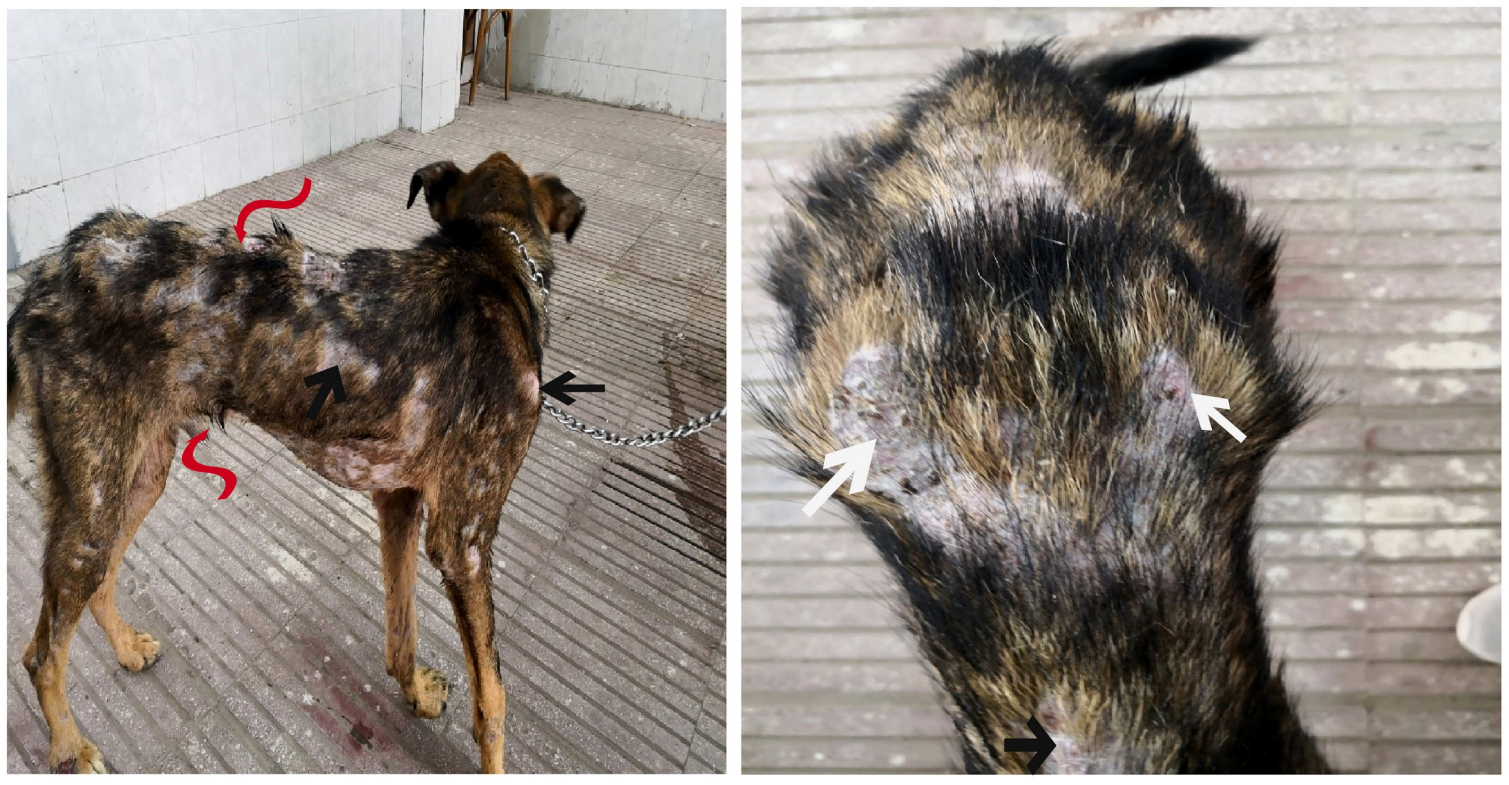
The image shows dogs in ORNIPURAL® treated group (Ornip^gr^) after 14 days following treatment with ORNIPURAL® (Ornip^gr^; day 28). Complete healing and disappearance of skin lesions (abscess, lacerations, and erosions) were observed (white arrows). The affected dogs were still suffering from poor health conditions, emaciation, and loss of body weight (curved red arrows). Areas of alopecia (Black arrows) were still appeared.

After treatment either with an herbal mixture of pomegranate peel extract and *C. longa* extract or with ORNIPURAL®, the dogs showed significant (*p* < 0.05) improvement in their body weights and appetite scores on days 21 and 28 (Herb^mix-gr^ or Ornip^gr^) when compared with their values in DexaHepato^gr^ (Days 7 and 14). However, this significant (*p* < 0.05) increase in investigated dogs’ body weights and appetite scores was higher in Herb^mix-gr^ than Ornip^gr^ on days 21 and 24. The examined dogs on day 21 after treatment with the herbal mixture (Herb^mix-gr^) for continuous 7 days showed a clear improvement in their activities and no lethargy with complete disappearance of alopecia, skin rashes, skin lacerations, and dehydration signs as well as complete healing of ruptured skin abscess (Figure 4). These findings contradict with those reported with Ornip^gr^ (Figure 5), which needed days of observation (day 28) more than those of herbal mixture until complete recovery and complete healing of skin lesions occurred. The affected dogs (Figure 5) were still suffering from poor health conditions, emaciation, and loss of body weight. Areas of alopecia, skin rashes, skin lacerations (ruptured skin abscesses), and skin abscesses have still appeared. In the examined dogs on day 28 after treatment with the herbal mixture (Herb^mix-gr^) for 14 continuous days, they restored their body conditions and body weights with the healthy appearance of their skin (Figure 6). In the Ornip^gr^ for 28 days and after a five-time treatment with ORNIPURAL®, complete healing and disappearance of skin lesions (Abscess, lacerations and erosions) were described on day 28; however, the dogs were still suffering from poor healthy conditions, emaciation, and loss of body weight. Furthermore, signs of dehydration and incomplete restoring of dogs’ normal gait and appetite were observed on day 28 in the ORNIPURAL® group (Ornip^gr^) (Table 1; Figure 7; Supplementary Table S2).

Temperature, pulse, and respiration showed no remarkable changes between Cont^gr^, Dexa_e_Hepato^gr^, Herb^mix-gr^, and Ornip^gr^ on days 0, 7, 14, 21, and 28. They were within their reference ranges (Table 1).

### 3.2 Whole blood picture indices

The study showed remarkable changes in whole blood picture indices in the examined dogs (Tables 2, 3). There was a significant (*p* < 0.05) reduction in red blood cells (RBCs) and hemoglobin (Hb) in DexaHepato^gr^ compared to their values in Cont^gr^, Herb^mix-gr^, and Ornip^gr^. RBCs and Hb concentrations were within the reference ranges in Cont^gr^, Herb^mix-gr^, and Ornip^gr^. At the same time, the dogs (anemic animals) investigated after experimental induction of hepatopathy in DexaHepato^gr^ had lower RBCs and Hb concentrations than their reference ranges. Packed cell volume (PCV) values were not remarkably changed between Cont^gr^, Herb^mix-gr^, DexaHepato^gr^, and Ornip^gr^ where they were within the reference ranges. Total leukocyte count (TLC) and neutrophil values showed remarkable (*p* < 0.05) elevations after experimental induction of hepatopathy in dogs in DexaHepato^gr,^ while lymphocytes were significantly (*p* < 0.05) reduced compared to their values in Cont^gr^, Herb^mix-gr^, and Ornip^gr^ and with their reference ranges. The leukogram picture was within the reference ranges in Cont^gr^, Herb^mix-gr,^ and Ornip^gr^ (Tables 2, 3).

**Table 2:** Mean values (M ± SD) of RBCs, Hb, PCV, and TLC in investigated dogs.

	RBCs (_X_10^12^/L) (5.5–8.5) (72)					Hb (g/L) (120–180) (72)					PCV (L/L) (0.39 ± 0.02) (73)					TLC (_X_10^9^/L) (11.35 ± 0.50) (78)				
	Day 0^#^	Day 7^*^	Day 14^*^	Day 21^¶^	Day 28^¶^	Day 0^#^	Day 7^*^	Day 14^*^	Day 21^¶^	Day 28^¶^	Day 0^#^	Day 7^*^	Day 14^*^	Day 21^¶^	Day 28^¶^	Day 0^#^	Day 7^*^	Day 14^*^	Day 21^¶^	Day 28^¶^
Cont^gr^ (*n* = 30)	6.31 ± 0.71^a^					145.34 ± 7.11^a^					0.41 ± 0.07^a^					8.95 ± 1.02^b^				
DexaHepato^gr^ (*n* = 29)		5.01 ± 0.43^bc^	4.52 ± 0.55^c^				106.47 ± 5.22^b^	101.22 ± 6.06^b^				0.37 ± 0.02^a^	0.38 ± 0.08^a^				13.23 ± 2.16^a^	15.81 ± 2.10^a^		
Herb^mix-gr^ (*n* = 14)				5.23 ± 0.21^b^	5.41 ± 0.19^b^				131.04 ± 2.44^a^	141.27 ± 8.06^a^				0.40 ± 0.01^a^	0.41 ± 0.02^a^				9.47 ± 0.94^b^	9.04 ± 0.48^b^
Ornip^gr^ (*n* = 14)				5.36 ± 0.33^b^	5.57 ± 0.38^b^				118.3 ± 4.41^b^	122.70 ± 2.61^b^				0.37 ± 0.01^a^	0.38 ± 0.03^a^				8.21 ± 0.82^b^	9.67 ± 1.13^b^

Cont^gr^: Control group. DexaHepato^gr^: Steroidal-induced hepatopathy group. Herb^mix-gr^: Herbal mixture-treated group. Ornip^gr^: ORNIPURAL^®^ treated group. RBCs: red blood corpuscles. Hb: hemoglobin. PCV: packed cell volume. TLC: total leucocytic count. ^#^Control. ^*^Dexamethasone treatment. ^¶^Herbal mixture or ORNIPURAL® treatment. ^abc^Means with different superscript letters in different sampling times were significantly different (*p* < 0.05) between Cont^gr^, DexaHepato^gr^, Herb^mix-gr^, and Ornip^gr^ in days 0, 7, 14, 21, and 28. Reference values according to Felman et al. (72); Reddy et al. (73); Atata et al. (78).

**Table 3:** Mean values (M ± SD) of neutrophils, lymphocytes, eosinophils, and basophils in investigated dogs.

	Neutrophils (%) (60–77) (57)					Lymphocytes (%) (8–38) (91)					Eosinophils (%) (2–10) (57)					Basophils (%) (0–1) (91)				
	Day 0^#^	Day 7^*^	Day 14^*^	Day 21^¶^	Day 28^¶^	Day 0^#^	Day 7^*^	Day 14^*^	Day 21^¶^	Day 28^¶^	Day 0^#^	Day 7^*^	Day 14^*^	Day 21^¶^	Day 28^¶^	Day 0^#^	Day 7^*^	Day 14^*^	Day 21^¶^	Day 28^¶^
Cont^gr^ (*n* = 30)	70.75 ± 1.30^b^					28.03 ± 1.35^a^					0.86 ± 0.06^a^					0.36 ± 0.11^a^				
DexaHepato^gr^ (*n* = 29)		85.44 ± 1.62^a^	86.22 ± 1.74^a^				13.48 ± 1.70^b^	12.74 ± 1.33^b^				0.76 ± 0.17^a^	0.70 ± 0.12^a^				0.32 ± 0.06^a^	0.34 ± 0.04^a^		
Herb^mix-gr^ (*n* = 14)				73.22 ± 2.00^b^	72.85 ± 2.83^b^				25.66 ± 1.62^a^	25.99 ± 2.03^a^				0.74 ± 0.06^a^	0.76 ± 0.03^a^				0.38 ± 0.06^a^	0.40 ± 0.09^a^
Ornip^gr^ (*n* = 14)				76.43 ± 1.98^a^	75.02 ± 2.14^a^				22.63 ± 0.58^a^	24.07 ± 0.58^a^				0.63 ± 0.08^a^	0.61 ± 0.17^a^				0.31 ± 0.03^a^	0.30 ± 0.03^a^

Cont^gr^: Control group. DexaHepato^gr^: steroid-induced hepatopathy group. Herb^mix-gr^: Herbal mixture-treated group. Ornip^gr^: ORNIPURAL® treated group. ^#^ Control. ^*^Dexamethasone treatment. ^¶^Herbal mixture or ORNIPURAL® treatment. ^abc^Means with different superscript letters in different sampling times were significantly different (*p* < 0.05) between Cont^gr^, DexaHepato^gr^, Herb^mix-gr^, and Ornip^gr^ in days 0, 7, 14, 21, and 28. According to Sastry (57); Khan et al. (91), reference values.

Except for Hb, the whole blood picture indices, including RBCs, PCV, TLC, and differential leukocyte count (DLC), showed no remarkable changes between Cont^gr^, Herb^mix-gr^, and Ornip^gr^. Hb concentrations were significantly (*p* < 0.05) reduced in Ornip^gr^ when they compared with their values at Herb^mix-gr^ (Tables 2, 3).

### 3.3 Liver functions indices

Serum concentrations of TP, albumin, and globulin, as well as Albumin/globulin (A/G) ratio, showed no remarkable changes either between Cont^gr^, DexaHepato^gr^, Herb^mix-gr^, and Ornip^gr^ on days 0, 7, 14, 21, and 28. Moreover, they were within their reference ranges (Table 4).

**Table 4:** Mean values (M ± SD) of liver functions indices part one (total protein, albumin, globulin, and A/G ratio) in investigated dogs.

	Total proteins (g/L) (54–71) (89)					Albumin (g/L) (23–31) (90)					Globulin (g/L) (27–44) (89)					A/G ratio (0.59–1.11) (90)				
	Day 0^#^	Day 7^*^	Day 14^*^	Day 21^¶^	Day 28^¶^	Day 0^#^	Day 7^*^	Day 14^*^	Day 21^¶^	Day 28^¶^	Day 0^#^	Day 7^*^	Day 14^*^	Day 21^¶^	Day 28^¶^	Day 0^#^	Day 7^*^	Day 14^*^	Day 21^¶^	Day 28^¶^
Cont^gr^ (*n* = 30)	62.33 ± 1.96^a^					30.09 ± 1.16^a^					32.24 ± 2.33^a^					0.93 ± 0.07^a^				
DexaHepato^gr^ (*n* = 29)		58.08 ± 1.74^a^	57.01 ± 2.11^a^				27.58 ± 2.16^a^	25.44 ± 1.94^a^				30.5 ± 2.16^a^	31.57 ± 2.31^a^				0.90 ± 0.04^a^	0.81 ± 0.08^a^		
Herb^mix–gr^ (*n* = 14)				59.04 ± 2.15^a^	60.29 ± 2.36^a^				27.63 ± 2.46^a^	28.12 ± 2.01^a^				31.41 ± 1.88^a^	32.17 ± 2.16^a^				0.88 ± 0.1^a^	0.87 ± 0.12^a^
Ornip^gr^ (*n* = 14)				56.04 ± 1.87^a^	58.04 ± 1.92^a^				24.03 ± 2.26^a^	27.61 ± 2.53^a^				32.01 ± 1.88^a^	30.43 ± 2.16^a^				0.75 ± 0.09^a^	0.91 ± 0.06^a^

Cont^gr^: Control group. DexaHepato^gr^: Steroid-induced hepatopathy group. Herb^mix-gr^: Herbal mixture-treated group. Ornip^gr^: ORNIPURAL® treated group. A/G: albumin/globulin ratio. ^#^ Control. ^*^Dexamethasone treatment. ^¶^Herbal mixture or ORNIPURAL® treatment. ^a^Means with different superscript letters in different sampling times were significantly different (*p* < 0.05) between Cont^gr^, DexaHepato^gr^, Herb^mix-gr^, and Ornip^gr^ on days 0, 7, 14, 21, and 28. Reference values according to Kaneko et al. (89) and Khan et al. (90).

Serum activities of AST, ALT, and ALP were significantly (*p* < 0.05) increased in DexaHepato^gr^ (days 7 and 14) compared to their values in Cont^gr^ (day 0). This significant elevation disappeared due to herbal therapy or due to ORNIPURAL® therapy. In contrast, their activities were remarkably (*p* < 0.05) reduced at Herb^mix-gr^ or Ornip^gr^, particularly on day 28 when they compared with their values in DexaHepato^gr^ (days 7 and 14). In contrast, although a significant decline in serum activities of liver enzymes at Herb^mix-gr^ or Ornip^gr^ was reported after herbal mixture therapy or after ORNIPURAL® treatment, respectively, on days 21 and 28, serum activities of AST, ALT, and ALP were still significantly (*p* < 0.05) higher than their values in Cont^gr^ (Day 0). Serum activities of AST, ALT, and ALP were within the reference ranges at each of Cont^gr^ and Herb^mix-gr,^ while they were higher than their reference ranges in DexaHepato^gr^. No remarkable changes were stated for AST, ALT, and ALP between Herb^mix-gr^ and Ornip^gr^ in days 21 or 28 (Table 5).

**Table 5:** Mean values (M ± SD) of liver functions indices part two (AST, ALT, ALP, total bilirubin, and direct bilirubin) in investigated dogs.

		AST (U/L) (23–66) (89)				ALT (U/L) (21–102) (89)					ALP (U/L) (20–156) (89)					Total bilirubin (μmol/L) (1.71–8.55) (89)						Direct bilirubin (μmol/L) (0.17–8.38) (89)				
		Day 0^#^	Day 7^*^	Day 14^*^	Day 21^¶^	Day 28^¶^	Day 0^#^	Day 7^*^	Day 14^*^	Day 21^¶^	Day 28^¶^	Day 0^#^	Day 7^*^	Day 14^*^	Day 21^¶^	Day 28^¶^	Day 0^#^	Day 7^*^	Day 14^*^	Day 21^¶^	Day 28^¶^	Day 0^#^	Day 7^*^	Day 14^*^	Day 21^¶^	Day 28^¶^
Cont^gr^ (*n* = 30)		38.55 ± 2.33^d^					44.61 ± 4.81^d^					104.05 ± 18.3^c^					7.35 ± 2.74^a^					2.22 ± 0.53^a^				
DexaHepato^gr^ (*n* = 29)			52.96 ± 8.22^b^	60.53 ± 10.27^a^				108.37 ± 7.46^a^	115.20 ± 9.39^a^				142.67 ± 15.83^b^	232.73 ± 29.68^a^				6.84 ± 2.91^a^	6.84 ± 2.75^a^				1.88 ± 0.41^a^	1.71 ± 0.71^a^		
Herb^mix-gr^ (*n* = 14)					56.61 ± 7.67^a^	44.90 ± 9.57^c^				76.16 ± 8.07^b^	57.33 ± 5.14^c^				92.1 ± 8.66^c^	64.8 ± 10.36^d^				7.18 ± 2.74^a^	7.35 ± 2.82^a^				1.37 ± 0.43^a^	1.88 ± 0.19^a^
Ornip^gr^ (*n* = 14)				58.22 ± 8.02^a^	46.06 ± 7.33^c^				69.78 ± 10.15^b^	51.73 ± 4.89^c^				100 ± 13^c^	66.51 ± 8.81^d^				7.35 ± 1.62^a^	7.87 ± 1.62^a^				2.22 ± 0.34^a^	1.88 ± 0.17^a^	

Cont^gr^: Control group. DexaHepato^gr^: Steroid-induced hepatopathy group. Herb^mix-gr^: Herbal mixture-treated group. Ornip^gr^: ORNIPURAL® treated group. AST: Aspartate aminotransferase. ALT: Alanine aminotransferase. ALP: Alkaline phosphatase. ^#^Control. ^*^Dexamethasone treatment. ^¶^Herbal mixture or ORNIPURAL® treatment. ^a-d^Means with different superscript letters in different sampling times were significantly different (*p* < 0.05) between Cont^gr^, DexaHepato^gr^, Herb^mix-gr^, and Ornip^gr^ in days 0, 7, 14, 21, and 28. Reference values according to Kaneko et al. (89).

Serum concentrations of total bilirubin and direct bilirubin showed no significant changes between Cont^gr^, DexaHepato^gr^, Herb^mix-gr^, and Ornip^gr^. Moreover, they were within their reference ranges (Table 5).

### 3.4 Kidney functions indices

Kidney function indicators showed remarkable alterations for serum concentrations of blood urea nitrogen (BUN) and creatinine (Cr), whereas significant (*p* < 0.05) elevations in serum levels of BUN and Cr were reported in Cont^gr^ and treated group (Herb^mix-gr^ or Ornip^gr^) compared with their values in DexaHepato^gr^. These significant changes disappeared between Herb^mix-gr^ and Ornip^gr^ (Table 6).

**Table 6:** Mean values (M ± SD) of kidney functions indicators in investigated dogs.

		BUN (mmol/L) (3.57–10) (89)					Cr (μmol/L) (44.2–132.6) (89)					
		Day 0^#^	Day 7^*^	Day 14^*^	Day 21^¶^	Day 28^¶^	Day 0^#^	Day 7^*^	Day 14^*^		Day 21^¶^	Day 28^¶^
Cont^gr^ (*n* = 30)		10.35 ± 1.12^a^					85.75 ± 5.31^a^					
DexaHepato^gr^ (*n* = 29)			7.64 ± 0.91^b^	7.45 ± 0.82^b^				72.49 ± 3.54^b^	63.95 ± 2.65^c^			
Herb^mix-gr^ (*n* = 14)					9.39 ± 1.06^a^	10.12 ± 0.85^a^					53.93 ± 5.3^d^	80.14 ± 4.42^a^
Ornip^gr^ (*n* = 14)					10.05 ± 1.14^a^	10.86 ± 1.06^a^				51.27 ± 8.84^d^	76.08 ± 6.01^a^	

Cont^gr^: Control group. DexaHepato^gr^: Steroid-induced hepatopathy group. Herb^mix-gr^: Herbal mixture-treated group. Ornip^gr^: ORNIPURAL® treated group. BUN: Blood urea nitrogen. Cr: Creatinine. ^#^Control. ^*^Dexamethasone treatment. ^¶^Herbal mixture or ORNIPURAL® treatment. ^a-d^Means with different superscript letters in different sampling times were significantly different (*p* < 0.05) between Cont^gr^, DexaHepato^gr^, Herb^mix-gr^, and Ornip^gr^ in days 0, 7, 14, 21, and 28—reference values according to Kaneko et al. (89).

### 3.5 Ultrasonographic findings

**Figure 8:**
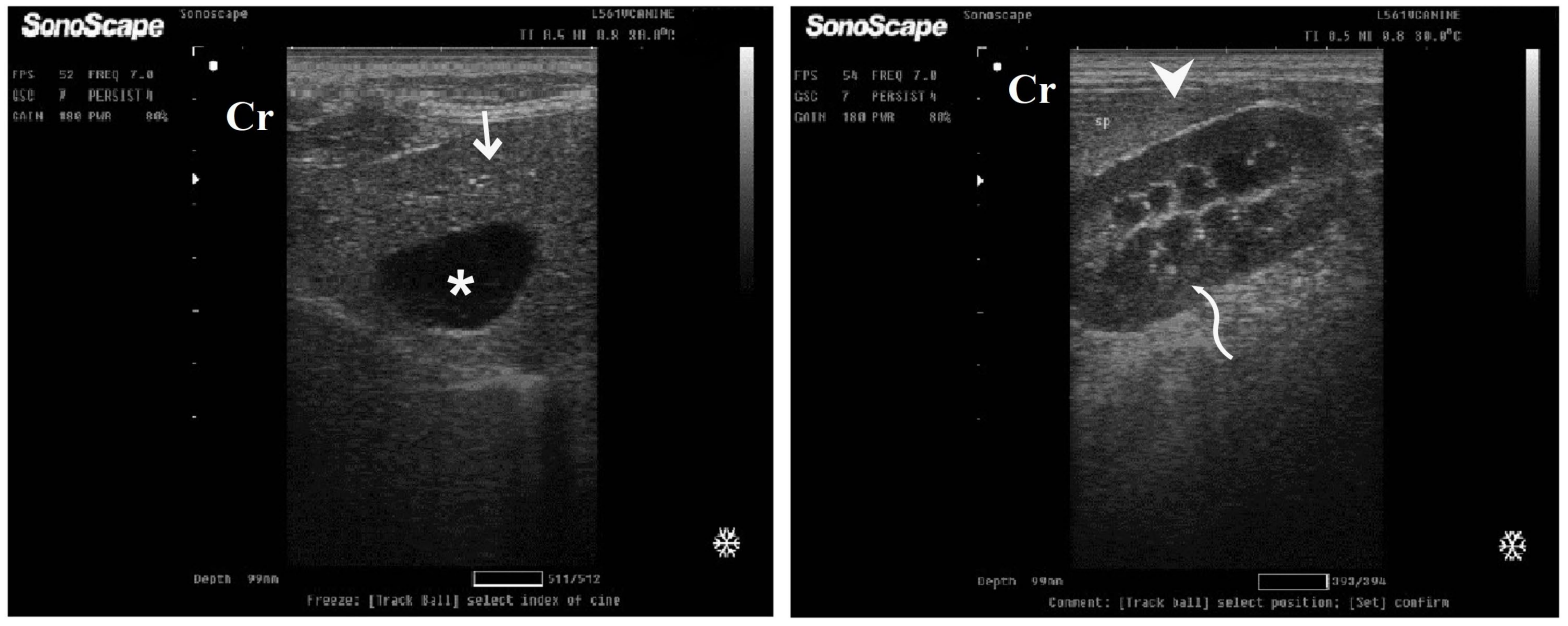
Ultrasonogram in a 3-year-old male dog in the control group (day 0) with healthy liver imaged through the left lateral recumbency, that is, intercostal approach (right 11^th^ ICSs) **(A)** or dorsal recumbency (subcostal approach) **(B)** using 7 MHz linear ultrasound transducer. It showed a normal ultrasonographic image of the hepatobiliary system. The normal hepatic parenchyma **(A)** has a uniform medium level of echogenicity, with interruption caused by the hepatic and portal veins (white arrows). The echotexture was coarser and more hypoechoic compared with the spleen (white head arrows) **(B)**. Compared with the kidney, the liver had an increased echogenicity (curved white arrows) **(B)**. The gall bladder **(A)** was anechoic and ovoid with a tapered neck (white asterisk). The wall of the gall bladder was not very clear at all. Cr: Cranial.

Cont^gr^ showed a standard ultrasonographic image of the hepatobiliary system and its vasculatures. The normal hepatic parenchyma has a uniform medium level of echogenicity, with interruption caused by the portal and hepatic veins. The echotexture was coarser and more hypoechoic compared with the spleen. In comparison with the spleen, the liver had a reduced echogenicity and a diffuse, slightly coarse-grained texture. Compared with the kidney, the liver had an increased echogenicity. Still, this relationship is highly variable as in some normal animals, the liver is isoechoic compared with the kidney, and in others, it is hypoechoic (Figure 8). Unless they were swollen, the bile ducts were invisible. The gall bladder was found when the scan was conducted ventrally toward the right side of the midline while the animal was in lateral recumbency. It was typically ovoid, tapering at the neck, and anechoic. The gall bladder wall was not visible in several instances and appeared as a skinny echogenic line (Figure 8).

**Figure 9:**
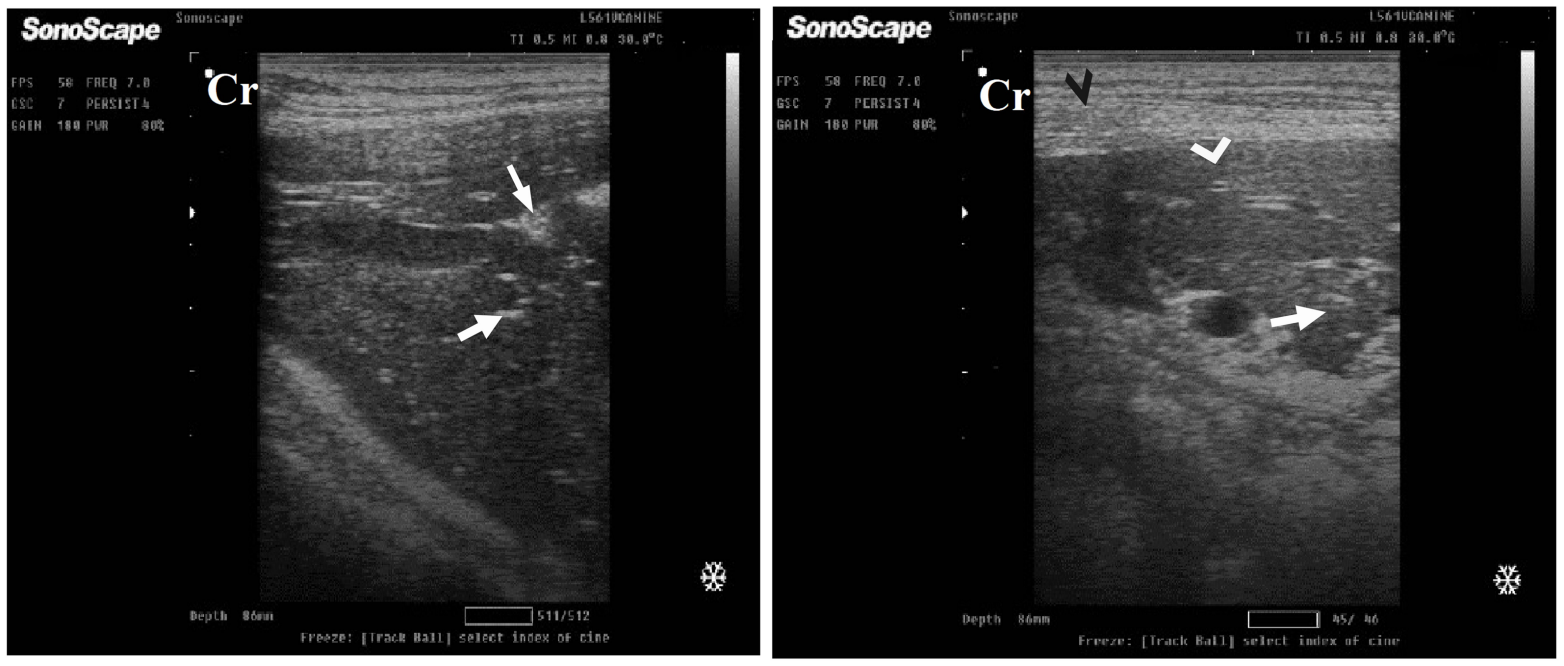
Ultrasonogram of longitudinal views of the liver in a 2-year-old male dog in a steroidal-induced hepatopathy group (DexaHepato^gr^) imaged through the dorsal recumbency (subcostal approach) using a 7 MHz linear ultrasound transducer. DexaHepato^gr^ (day 7) showed a slight increase in echogenicity of the hepatic parenchyma (white head arrows), but it was still less than that of the spleen (black head arrows). The liver showed a focal hyperechoic area (white arrows) within the parenchyma. The hepatic parenchyma had a heterogeneous nature. Cr: Cranial.

**Figure 10:**
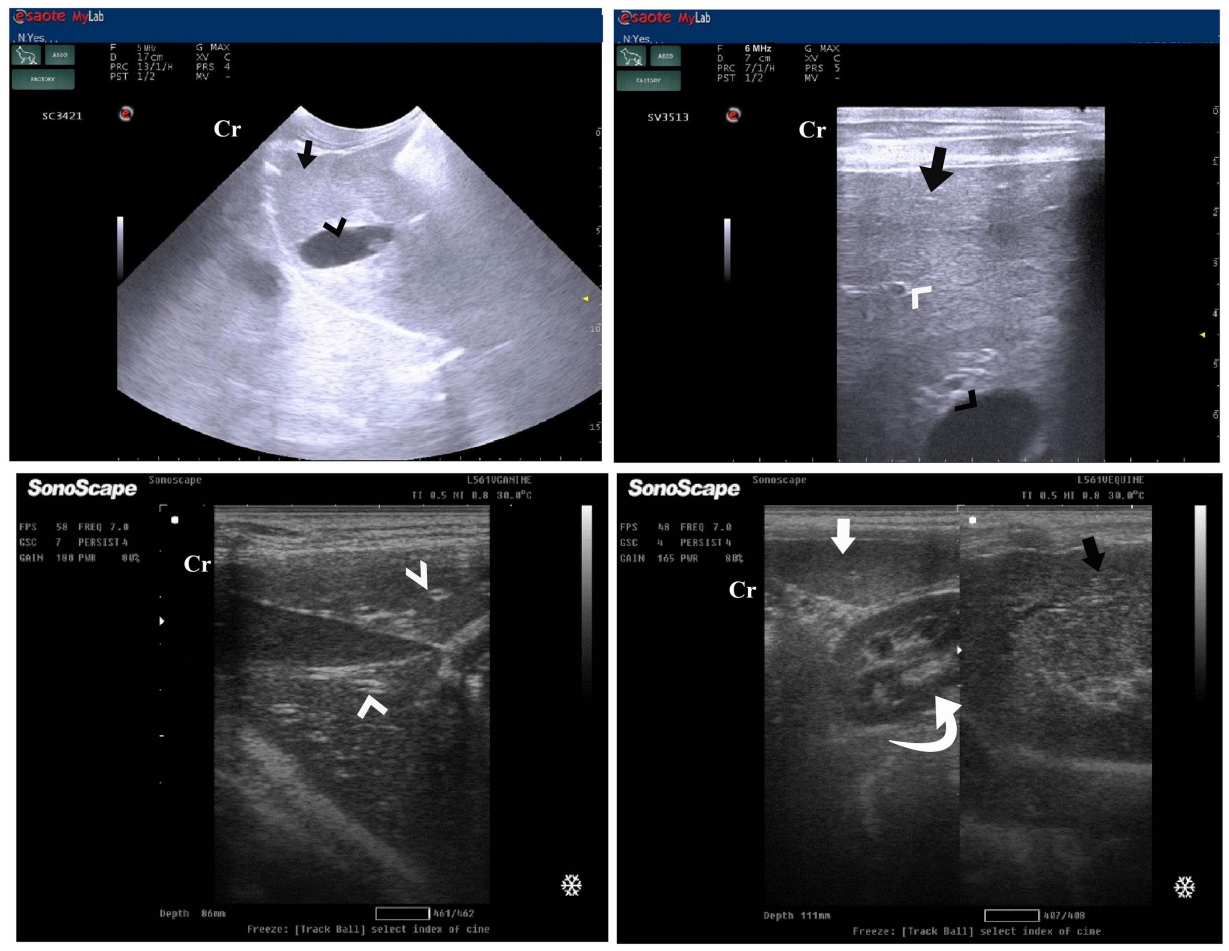
Ultrasonogram of transverse views **(A,B)** and longitudinal views **(C,D)** and of the liver in a 2-year-old female dog in a steroidal-induced hepatopathy group (DexaHepato^gr^) imaged through the dorsal recumbency (subcostal approach) using 6 and 7 MHz linear ultrasound transducer or 5 MHz micro convex ultrasound. DexaHepato^gr^ on day 14 showed a diffuse increase in hepatic parenchymal echogenicity (black arrows) as it was hyperechoic in comparison to the renal cortex (white curved arrows) and was like echogenicity of splenic parenchyma (white arrows). The diffuse and homogenous hyperechogenicity of the hepatic parenchyma caused indistinct visualization of the hepatic vessel wall. The hepatic parenchyma sometimes appeared heterogeneous and contained hypoechoic foci, most likely due to concurrent nodular hyperplasia (white head arrows). The gall bladder (black head arrows) was anechoic and ovoid with a tapered neck. The wall of the gall bladder was a thin echogenic line. Cr: Cranial.

DexaHepato^gr^ on day 7 in both the herbal mixture and ORNIPURAL® groups showed a slight increase in echogenicity of the hepatic parenchyma. However, it was still less than the spleen reported ultrasonographically. The liver showed a focal hyperechoic area within the liver parenchyma (Figure 9). DexaHepato^gr^ on day 14 showed a diffuse increase in hepatic parenchymal echogenicity as it was hyperechoic in comparison to the renal cortex and was like echogenicity of splenic parenchyma. The diffuse and homogenous hyperechogenicity of the hepatic parenchyma associated with hepatopathy in affected dogs induced indistinct visualization of hepatic vasculature walls, and thus, it was difficult to detect. The hepatic parenchyma sometimes appeared heterogeneous and contained hypoechoic foci, most likely due to concurrent nodular hyperplasia (Figure 10).

**Figure 11:**
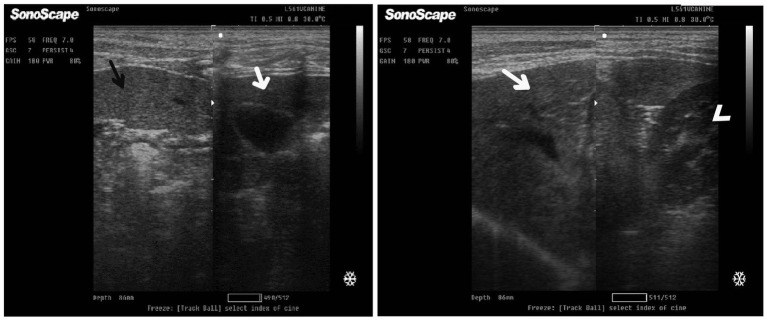
Ultrasonogram of longitudinal views of the liver in a 2-year-old female dog in a herbal mixture-treated group (Herb^mix-gr^; day 21) imaged through the dorsal recumbency (subcostal approach) using 7 MHz linear ultrasound transducer. It showed normal hepatic parenchyma as a uniform medium level of echogenicity (white arrows). Compared with the spleen, the liver should have a reduced echogenicity (black arrows). Compared with the kidney, the liver had an increased echogenicity (white head arrows). Cr: Cranial.

**Figure 12:**
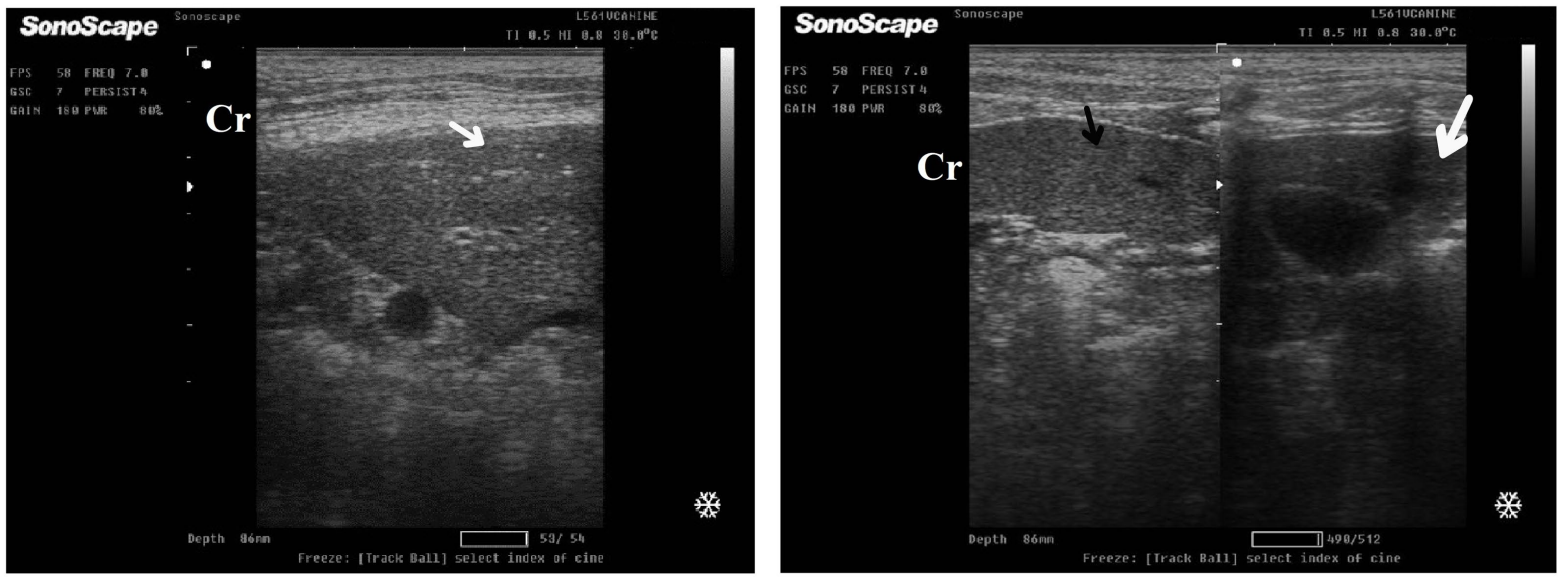
Ultrasonogram of longitudinal views of the liver in a 4-year-old male dog in an herbal mixture-treated group (Herb^mix-gr^; day 28) imaged through the dorsal recumbency (subcostal approach) using 7 MHz linear ultrasound transducer. It showed normal hepatic parenchyma as a uniform medium level of echogenicity (white arrows). The liver parenchyma had reduced echogenicity compared to the spleen (black arrows). Cr: Cranial.

After 7 days of treatment with an herbal mixture of *C. longa* extract and pomegranate peel extract, hepatic ultrasonography of Herb^mix-gr^ on day 21 of the current study revealed that liver parenchyma returned to normal, the hepatic parenchyma appeared as uniform homogenous numerous echoes. The normal hepatic parenchyma had a uniform medium level of echogenicity, with interruption caused by the hepatic and portal veins. The liver had a reduced echogenicity and a diffuse, slightly coarse-grained texture compared to the spleen. Compared with the kidney, the liver had an increased echogenicity (Figure 11). The same ultrasonographic findings (Figure 12) were reported for the liver at Herb^mix-gr^ (day 28).

**Figure 13:**
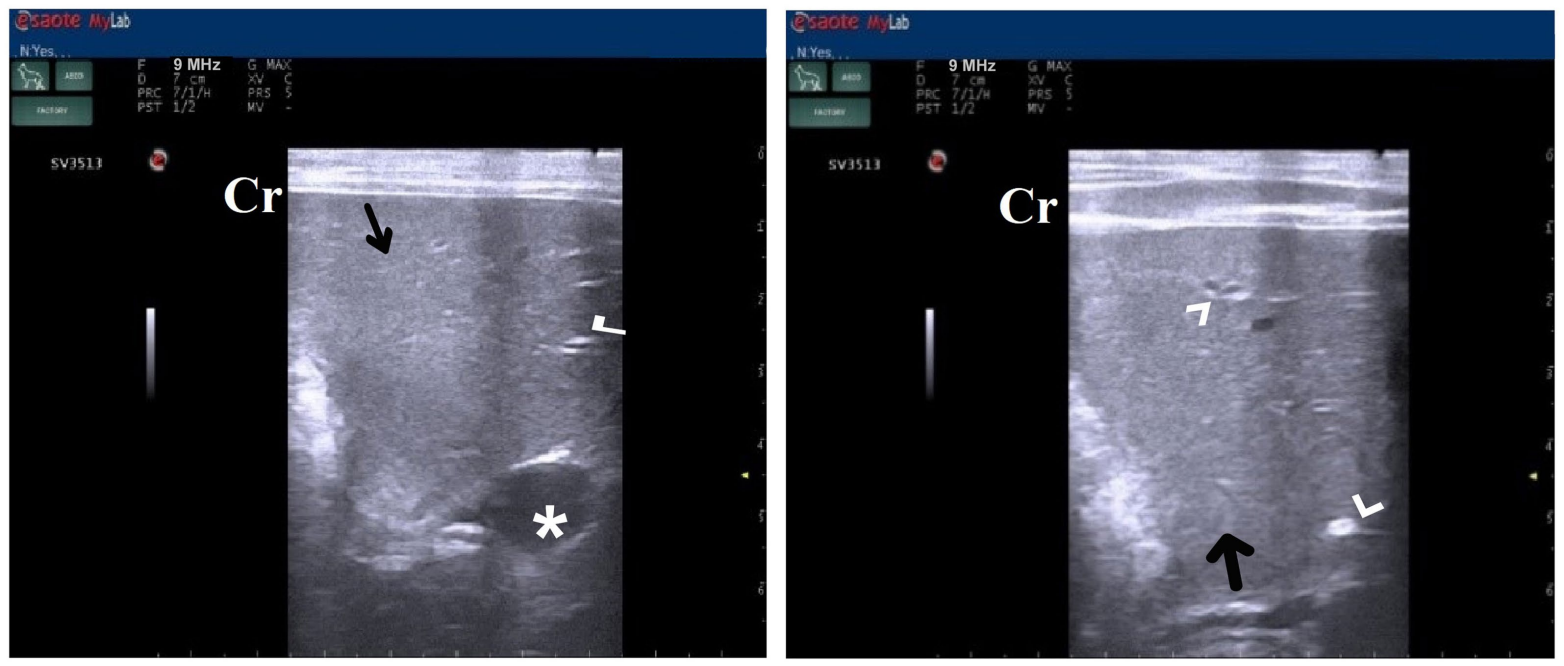
Ultrasonogram of longitudinal views of the liver in a 3-year-old male dog in an ORNIPURAL^®^-treated group (Ornip^gr^; day 21) imaged through the dorsal recumbency (subcostal approach) using a 9 MHz linear ultrasound transducer. It showed a diffuse increase in hepatic parenchymal echogenicity (black arrows). The diffuse and homogenous hyperechogenicity of the hepatic parenchyma caused indistinct visualization of the hepatic vessel wall. The hepatic parenchyma sometimes appeared heterogeneous and contained hypoechoic foci, most likely due to concurrent nodular hyperplasia (white head arrows). The gall bladder (white asterisk) was anechoic and ovoid with a tapered neck. The wall of the gall bladder was fragile and was not clear. Cr: Cranial.

After 7 days of treatment with ORNIPURAL®, in the Ornip^gr^ on day 21 of the current study, there was a diffuse increase in hepatic parenchymal echogenicity as it was hyperechoic in comparison to renal cortex and was like echogenicity of splenic parenchyma. The diffuse and homogenous hyperechogenicity of the hepatic parenchyma made hepatic vessel walls indistinct and difficult to detect. The hepatic parenchyma sometimes appeared heterogeneous and contained hypoechoic foci, most likely due to concurrent nodular hyperplasia (Figure 13).

**Figure 14:**
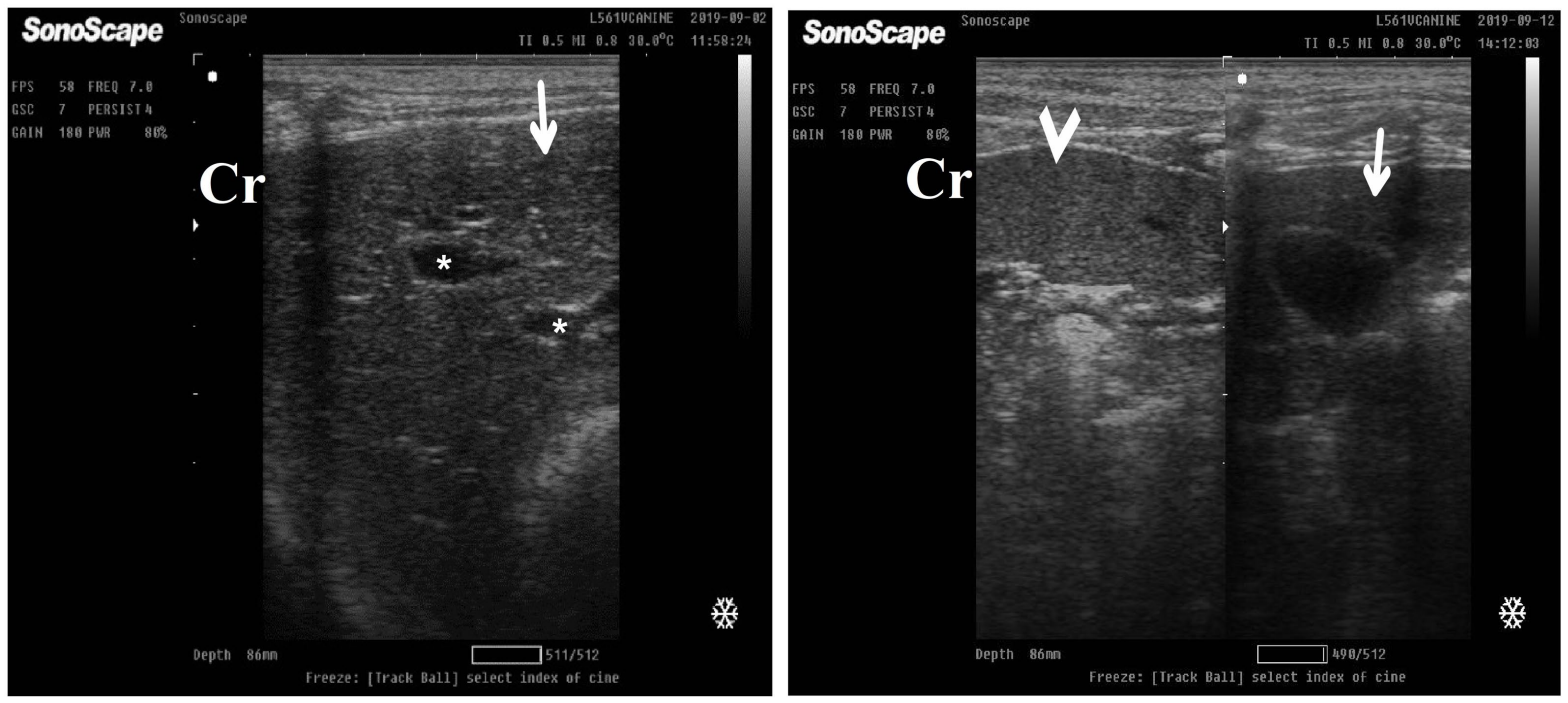
Ultrasonogram of longitudinal views of the liver in a 3-year old s-male dog in an ORNIPURAL® treated group (Ornip^gr^; day 28) imaged through the dorsal recumbency (subcostal approach) using a 7 MHz linear ultrasound transducer. The liver parenchyma became slightly normal as it had uniform homogenous numerous echoes (white arrows), with interruption caused by the hepatic and portal veins. The hepatic vasculatures (white asterisk) were relatively imaged. However, the hepatic parenchyma echogenicity was like the echogenicity of splenic parenchyma (white head arrows). Cr: Cranial.

After 14 days of treatment with ORNIPURAL^®^, hepatic ultrasonography of Ornip^gr^ on day 28 of the current study showed that liver parenchyma became slightly normal as it had uniform homogenous numerous echoes, with interruption caused by the hepatic and portal veins. The hepatic vasculatures were relatively imaged. However, the hepatic parenchyma showed an increase in its echogenicity as it was hyperechoic compared to the renal cortex and was like the echogenicity of splenic parenchyma (Figure 14).

### 3.6 Histopathological findings

**Figure 15:**
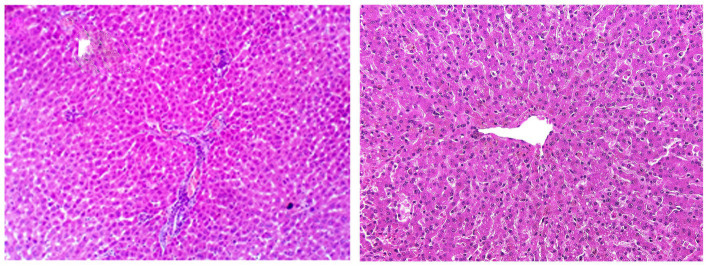
Photomicrographs of the liver in a healthy dog (Day 0) showing normal of most hepatic parenchyma.

Table 7 summarizes the primary histopathological liver lesions among different experimental groups. In the control group on day 0, either in the herbal mixture group or the ORNIPURAL® group, the hepatic parenchyma contains plates and sinusoids, which are usually one cell thick and have a regular arrangement around the central hepatic vein, causing a radiating pattern of the sinusoidal network. Outside the perivenular zone, the plates are less regularly arranged, with a loss of the radiating pattern. Individual hepatocytes are polygonal, pale to eosinophilic cytoplasm, and clearly outlined cell margins. The nucleus is centrally placed, and one or more nucleoli may be recognized. Hepatic plates are separated by vascular sinusoids formed from specialized endothelial cells, evident only by their flattened nuclei along the sinusoids (Figure 15).

**Table 7:** The main histopathological lesions of the liver in investigated dogs.

	Hydropic degenerations			Steatosis			Necrotic hepatocytes			Round cells infiltrations			Dilated hepatic blood vessels and sinusoids			Hemorrhage		
	Day 0^#^	Day 14^*^	Day 28^¶^	Day 0^#^	Day 14^*^	Day 28^¶^	Day 0^#^	Day 14^*^	Day 28^¶^	Day 0^#^	Day 14^*^	Day 28^¶^	Day 0^#^	Day 14^*^	Day 28^¶^	Day 0^#^	Day 14^*^	Day 28^¶^
Cont^gr^ (*n* = 30)	X			X			X			X			X			X		
DexaHepato^gr^ (*n* = 29)		√√√			√√√			√√			√			√√			√√	
Herb^mix-gr^ (*n* = 14)			X			X			X			√			√			X
Ornip^gr^ (*n* = 14)			X			X			X			X			√			X

Cont^gr^: Control group. DexaHepato^gr^: Steroidal-induced hepatopathy group. Herb^mix-gr^: Herbal mixture-treated group. Ornip^gr^: ORNIPURAL® treated group. ^#^Control. ^*^Dexamethasone treatment. ^¶^Herbal mixture or ORNIPURAL® treatment. X: No changes. √: Mild changes. √√: Moderate changes. √√√: Severe changes.

**Figure 16:**
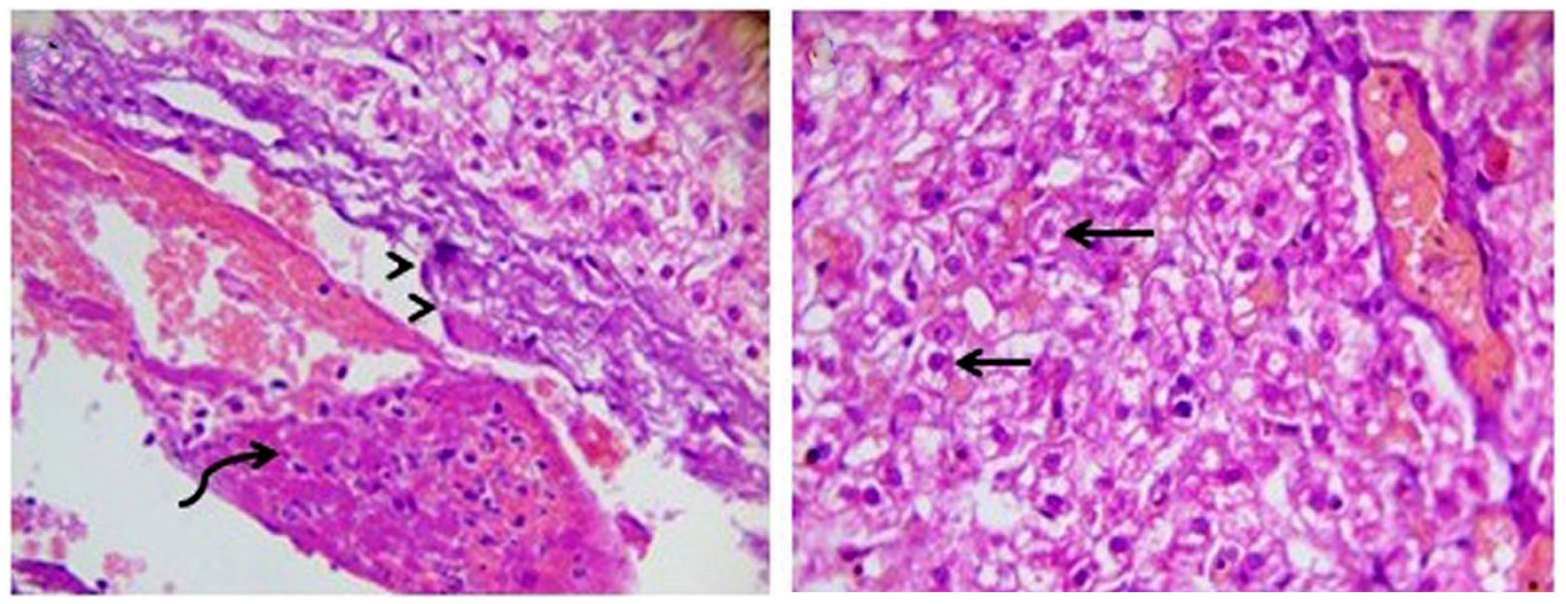
Photomicrograph of the liver in a dog at steroidal-induced hepatopathy group (DexaHepato^gr^ day 14) showing perivascular hydropic degenerations within hepatic parenchyma (black arrows) and thrombus formation (curved black arrows) within hepatic blood vessels beside necrotic tunica intema (head black arrows). [H&E X 400].

DexaHepato^gr^ on day 14 in each of the herbal mixture group and ORNIPURAL® group showed histopathological changes in the liver of dogs with steroidal-induced hepatopathy. In contrast, the plate showed hydropic degenerations within hepatic parenchyma (55%) and thrombus formation within the hepatic blood vessels adjacent to necrotic tunica intima (Figure 16).

**Figure 17:**
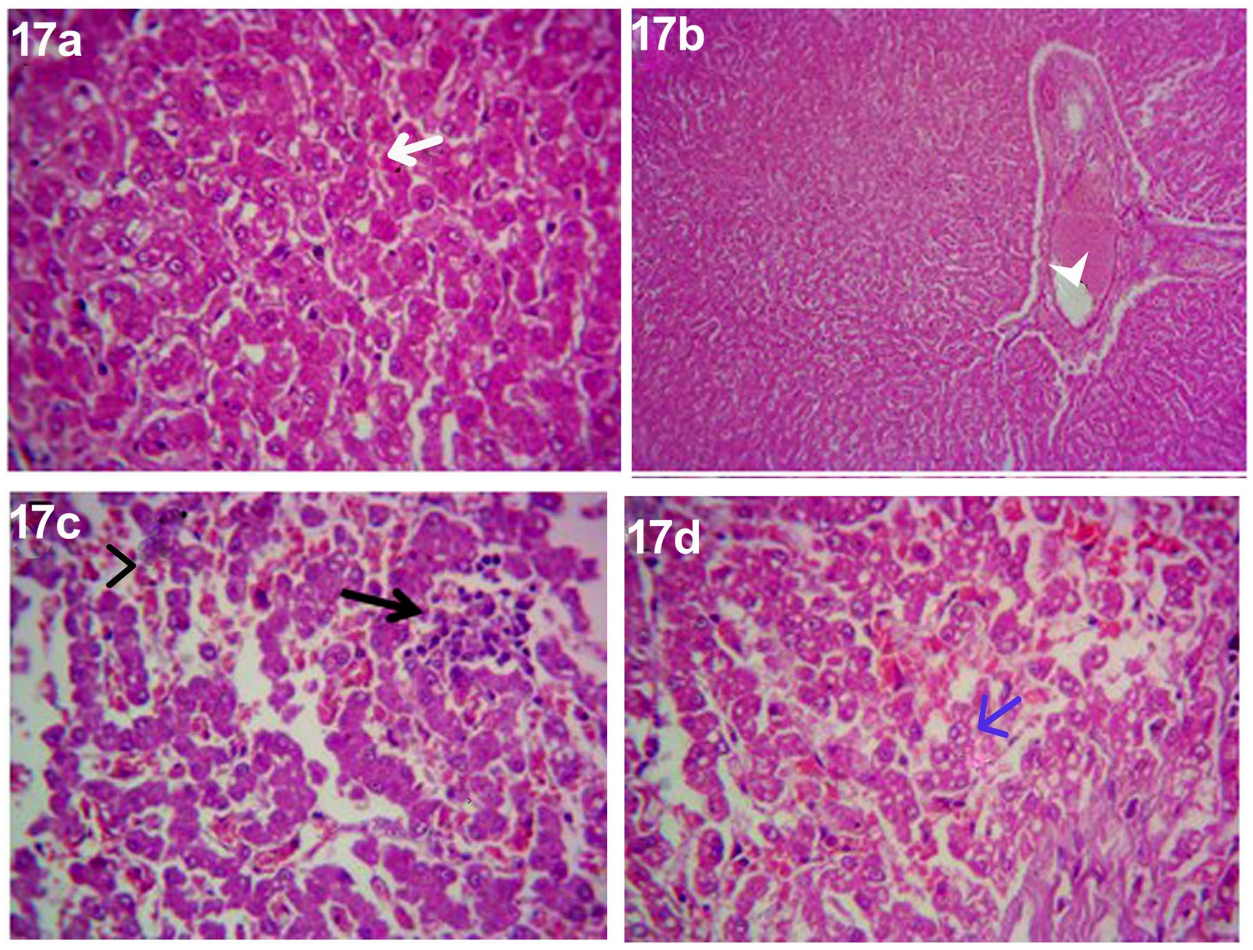
Photomicrograph of the liver in a dog in an herbal mixture-treated group (Herb^mix-gr^; day 28) showing normal of most hepatic parenchyma (White arrows), focal necrotic area infiltrated with mononuclear cells (Black arrows), microsteatosis in some hepatocytes (blue arrows) and dilated hepatic blood vessels (white head arrows) and sinusoids (black head arrows) [H&E X100 for **(a)**; H&E X400 for **(b–d)**].

After 14 days of treatment with an herbal mixture of *C. longa* extract and pomegranate peel extract, Herb^mix-gr,^ displayed histopathological changes in the liver sections on day 28. In contrast, the plate showed that majority of the hepatic parenchyma appeared normal. However, the focal necrotic area infiltrated with mononuclear cells associated with microsteatosis in some hepatocytes (15%) was visualized. Dilated hepatic blood vessels and dilated sinusoids were also encountered (Figure 17).

**Figure 18:**
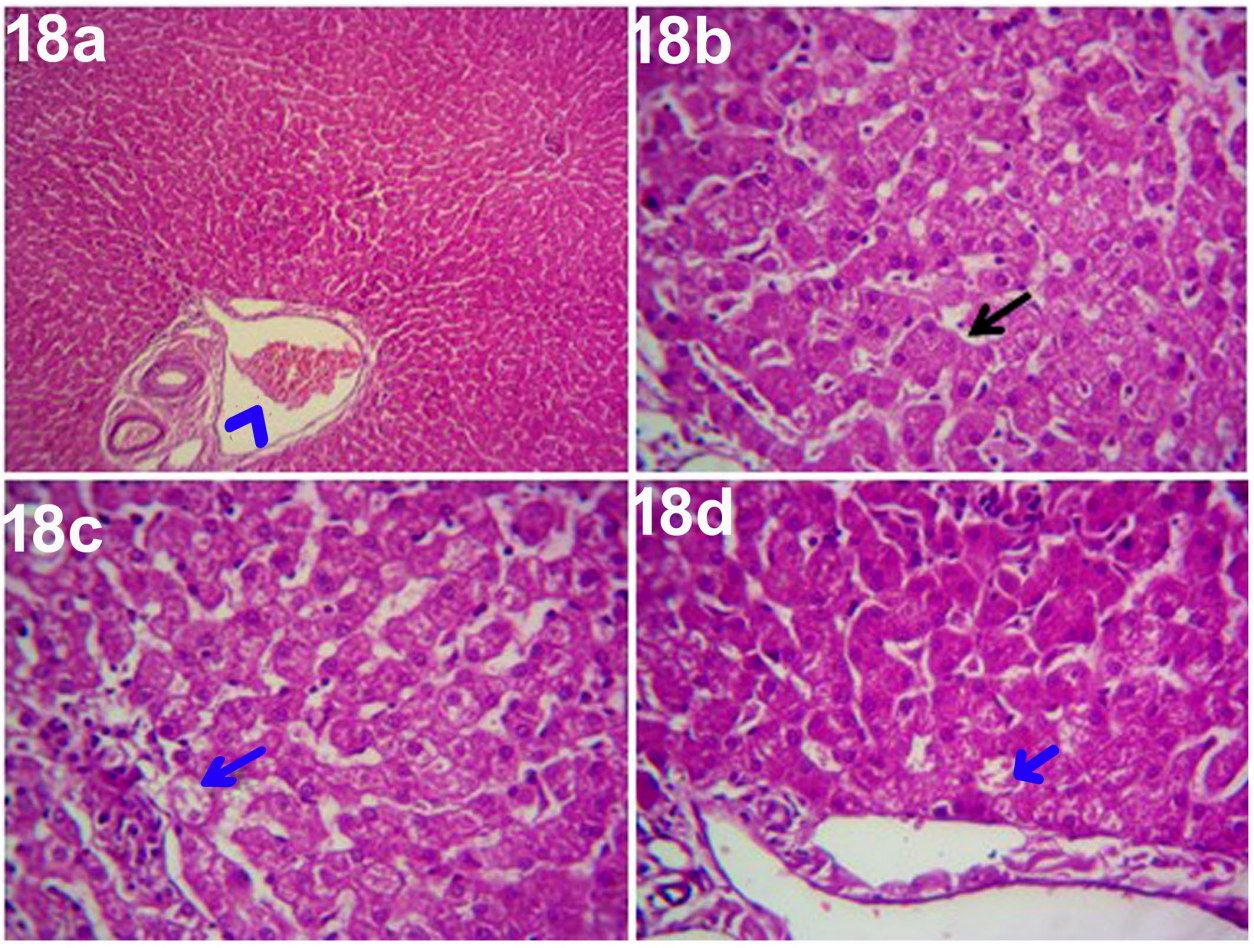
Photomicrograph of the liver in a dog at ORNIPURAL® treated group (Ornip^gr^; day 28) showing normal hepatic parenchyma (black arrows), unicellular necrotic changes of some hepatocytes (blue arrows) and congestion of portal vein (Blue head arrows) [H&E X100 for **(a);** H&E X400 for **(b–d)**].

The histopathological findings of hepatic parenchyma in steroidal-induced hepatopathy dogs after 14 days of treatment with ORNIPURAL® at Ornip^gr^ (Day 28) showed normal hepatic parenchyma. However, unicellular degenerative changes (10%), necrotic changes of some hepatocytes, and congestion of some portal veins were also noticed (Figure 18).

## 4 Discussion

### 4.1 Clinical findings

Clinical signs were only recognized at an advanced stage where the hepatocyte damage became severe, and treatment at this stage was less effective (1, 2, 4). The present study showed that the control dogs on day 0 had normal findings and suitable body weights. Their vital signs, including temperature, pulse, and respiratory rate, were within the reference values reported by Sturgess (49) and Sastry (57). Other reports added that anorexia, depression, lethargy, vomiting, diarrhea, bleeding tendencies, jaundice, weight loss, polydipsia, polyuria, ascites, and melena were typical clinical signs of liver disease (4, 9). Jaundice, anorexia, weight loss, nausea, abdominal pain, vomiting, and dehydration were the most observed signs in dogs with cholelithiasis (1, 5).

Steroid hepatopathy was a condition that only affected dogs. It usually appeared 2 to 3 days after using corticosteroids and was characterized by changes in the morphology and function of the liver (4). DexaHepato^gr,^ in the current study after dexamethasone injection revealed dramatic changes in the clinical findings of the investigated dogs as well as lowering in their body weights and appetite scores were reported. The severity of clinical findings in DexaHepato^gr^ was more evident on day 14 than on day 7. Skin lacerations and ruptured dermal abscesses were seen in DexaHepato^gr^ on day 14. These findings were in agreement with Eman et al. (4), Tilley and Smith (58), and Abdou et al. (59). Abdou et al. (59) described further clinical findings associated with prolonged dexamethasone overdosing, including depression, polyuria, polydipsia, muscle weakness, polyphagia, panting, abdominal distention, and prostration. Plump (60) mentioned that muscle wasting, ataxia, and lethargy could be attributed to the direct effect of glucocorticoids on muscles. On the other side, these dermatological changes, such as the development of skin abscesses, skin rashes, and alopecia, might be owned to long-term steroid use, which caused atrophy of the skin, hair follicles, and pilosebaceous apparatus. Skin infections were shared due to dermal and decreased immunity; these findings were in agreement with Gouda (61). Davies (62) and Negasee (63) stated non-specific signs related to acute hepatitis, which included waxing and waning gastrointestinal signs, anorexia, weight loss, depression, lethargy, polydipsia, and polyuria. In contrast, specific clinical signs of acute cholecystitis were reported by Kumar and Srikala (7), Kumar et al. (13), and Negasee (63), which that included anorexia, lethargy, vomiting, abdominal pain, and fever, mild to moderate jaundice and ileus. Many prescription drugs, such as steroids, cause liver disease in modern life, causing serious health problems (8, 30, 63). Glucocorticoids were used in dogs to treat inflammation and immunosuppression, but they had serious side effects when used for long periods (64).

Medicinal plants played an important role because they maintained health and cured various diseases, including liver disorders, without causing toxicity. Nowadays, more than half of all modern drugs in clinical use are derived from natural products (23). After treatment either with a herbal mixture of pomegranate peel extract and *C. longa* extract or with ORNIPURAL®, the dogs showed significant improvement in their body weights and appetite scores on days 21 and 28 when they compared with their values in DexaHepato^gr^. However, this substantial improvement was more significant in Herb^mix-gr^ than Ornip^gr^. Paolinelli et al. (32) reported that turmeric, a powder derived from *C. longa* plant, inhibited lipid peroxidation, a degenerative process involving free radicals and polyunsaturated fatty acids, mainly arachidonic. The peroxidative effect on polyunsaturated fatty acids caused hepatic pathogenesis; turmeric had been shown to be beneficial in reducing body weight. In the current study, the investigated dogs at Herb^mix-gr^ (day 21) and after treatment with herbal mixture displayed clear improvement in their activities and no lethargy with complete disappearance of alopecia, skin rashes, skin lacerations, and signs of dehydration as well as complete healing of ruptured skin abscess. These findings contradict those reported with Ornip^gr^, which needed more days of observation than those required by a herbal mixture until full recovery and healing of skin lesions were achieved.

Furthermore, signs of dehydration and incomplete restoring of dogs’ normal gait and appetite were observed on day 28 in Ornip^gr^. The previous studies mentioned that turmeric had long been known for its various medicinal properties, such as antiseptic, antibacterial, and anti-inflammatory, as well as its use in the treatment of digestive disorders, gastric upset, and liver and kidney disease. It stimulates bile production, improving digestion and eliminating toxins from the liver (34, 65–68). The anti-inflammatory properties of turmeric lowered the production of inflammation-inducing histamine, extending the action of the natural anti-inflammatory adrenal hormone cortisol and finally improving circulation by flushing toxins out of small joints where cellular wastes and inflammatory compounds were trapped. Turmeric’s hepatoprotective properties were primarily due to its antioxidative properties and ability to reduce the formation of proinflammatory cytokines. Turmeric, *C. longa*, and *curcumin* were used to treat biliary hyperplasia, fatty changes, and necrosis (30, 34, 67). Pomegranate is high in carbohydrates, minerals, crude fiber, and various biologically active compounds such as vitamin C and phenolic compounds such as punicalagin and ellagic acid, known as natural antioxidants (69). It had been reported that pomegranate phenolic compounds and vitamin C had antioxidant and free radical scavenging activity. Compared to the control, pomegranate significantly affected some oxidant and antioxidant enzymes in the liver (35). Pomegranate fruits and peels were used to treat cardiovascular disorders, had neuroprotective activity, a hypoglycemic effect, anti-cancer properties against prostate and colon cancer, antioxidant properties, and hepatoprotective properties (70).

Temperature, pulse, and respiration showed no remarkable changes between Cont^gr^, DexaHepato^gr^, DexaHepato^gr^, Herb^mix-gr^, and Ornip^gr^. Their vital signs, including temperature, pulse, and respiratory rate, were within the reference values reported by Sturgess (49) and Sastry (57). In liver diseases, *C. longa*’s potential ability to inhibit nuclear factor-kappa B, a modulator of several proinflammatory and profibrotic cytokines and its antioxidant properties, provided a pharmacological basis for its use. In liver diseases, *C. longa*, like silymarin, is a potent hepatoprotective and reduces hepatic damage in various acute and chronic liver injury models (33). Pomegranate peel reduces lipid peroxidation and nitric acid in the liver and kidney and has strong antioxidative properties, anti-inflammatory properties, anti-cancer activity, and anti-atherosclerotic effects. Pomegranate has hepatoprotective properties (35).

### 4.2 Whole blood picture indices

Sen et al. (12) added that a two-year-old Labrador Retriever bitch with ascites of hepato-cardiac origins had regular hemato-biochemical reports except TLC. These findings supported the current study and showed remarkable changes in whole blood picture indices. A significant reduction in RBCs and Hb in DexaHepato^gr^ compared to their values in Cont^gr^. These results contradicted McKay and Cidlowski (71), revealing that corticosteroids raised hemoglobin and RBCs in the blood in the first week, possibly by slowing erythrophagocytosis. Owing to herbal therapy or ORNIPURAL® treatment in the present study, RBCs and Hb concentrations were remarkably increased at Herb^mix-gr^ or Ornip^gr^ compared to their values in DexaHepato^gr^. RBCs and Hb concentrations were within the reference ranges in Cont^gr^, Herb^mix-gr^, and Ornip^gr.^ At the same time, the investigated dogs (anemic animals) after experimental induction of hepatopathy in DexaHepato^gr^ had lower RBCs and Hb concentrations than their reference ranges that were reported by Felman et al. (72) and Reddy et al. (73). Pomegranate is an anthelmintic, antidiarrheal agent with antioxidant activity and a practical effect against liver fibrosis (40).

On the other hand, anemia reported in hepatic affections, usually normocytic and normochromic, was related to inefficient use of systemic iron stores (74, 75). Moreover, anemia in dogs with liver diseases might be associated with RBC fragility, which results from high levels of bile acids (76, 77). Dogs treated with glucocorticoids showed an increase in total RBC count and PCV in the first week of dexamethasone injection, and this increase of RBCs might be due to retardation of erythrophagocytosis, polycythemia allowed increase of PCV% due to decrease in plasma protein. After the second week, the level of RBCs and PCV showed a significant sharp decrease (61, 75).

Regarding PCV values, they were not remarkably changed between Cont^gr^, Herb^mix-gr^, DexaHepato^gr^, and Ornip^gr^ where they were within the reference ranges that were reported by Sudhakara Reddy et al. (73) and Atata et al. (78). TLC and neutrophil values showed a remarkable elevation after experimental induction of hepatopathy in dogs in DexaHepato^gr.^ At the same time, lymphocytes were significantly reduced compared with their values in Cont^gr^, Herb^mix-gr^, and Ornip^gr^ and their reference ranges reported by Felman et al. (72) and Atata et al. (78). The previous studies added that leukocytosis was observed in dogs given an overdose of glucocorticoids, as well as neutrophilia, lymphopenia, and eosinopenia, as indicators of the immunosuppressive effect of glucocorticoids (79). Moreover, glucocorticoid treatment caused an increase in polymorphonuclear leukocytes in blood due to a faster rate of entry from the marrow and a slower rate of removal from the vascular compartment.

In contrast, after glucocorticoid administration, the number of lymphocytes, eosinophils, monocytes, and basophils decreased (71). Except for Hb, the whole blood picture indices, including RBCs, PCV, TLC, and DLC, showed no remarkable changes between Cont^gr^, Herb^mix-gr^, and Ornip^gr^. Hb concentrations significantly decreased in Ornipgr when compared with their values at Herb^mix-gr^. On the other hand, abnormalities in hematology analysis in animals with gall bladder issues might involve non-regenerative anemia or neutrophilic leukocytosis with or without a left shift, and toxic changes were usually seen (80, 81). Hemograms in animals with hepatobiliary disorders exhibited anemia or erythrocyte morphologic abnormalities (82, 83), while cholelithiasis animals revealed a normal haemogram except for leukocytosis with neutrophilia (8, 12, 84). Complete blood counts were usually unremarkable in dogs with gallbladder mucocele, with leukocytosis evident in 46.9% of the cases. The leukocytosis can be characterized by left-shift neutrophilia (regenerative or degenerative) (85, 86).

### 4.3 Liver function indices

Dogs with liver disease could be subclinical for a long time. Furthermore, since these subclinical animals often had regular biochemical examinations, they were difficult to diagnose using current screening methods (3, 4). Glucocorticoids caused hepatic glycogen accumulation and increased induced and intracellular liver enzymes in dogs. This type of hepatopathy was typically reversible (87, 88). Unfortunately, several complications have been recorded as a result of corticosteroid use. Steroid hepatopathy was one of the most common pathologies in dogs treated with corticosteroids (16, 88). The present results revealed that serum concentrations of TP, albumin, and globulin, and A/G ratio showed no remarkable changes between Cont^gr^, DexaHepato^gr^, Herb^mix-gr^, and Ornip^gr^. Moreover, they were within the reference ranges that were stated by Elhiblu et al. (75), Kaneko et al. (89), and Khan et al. (90).

In contrast, because the liver was the primary site for protein synthesis and degradation, hypoproteinemia was the most common finding in chronic disorders such as cirrhosis and portosystemic vascular abnormalities. Low serum albumin concentrations as a result of liver disease indicated diffuse and chronic hepatopathies. Hypoalbuminemia might also be caused by decreased nutrient uptake due to hepatopathies (75). Cirrhosis has been shown to impair albumin and prothrombin synthesis (83). Salama et al. (30) stated that the ethanol extract of *C. longa* rhizomes is a promising therapy for treating liver cirrhosis. In contrast, an improvement in the level of TP in rats was reported. Sen et al. (12) described standard biochemical profiles in ascites of hepato-cardiac origin in a two-year-old Labrador Retriever bitch.

Serum activities of AST, ALT, and ALP significantly increased in DexaHepato^gr^ compared to their values in Cont^gr^. This significant elevation disappeared due to herbal therapy or due to ORNIPURAL® therapy. In contrast, their serum activities were remarkably reduced in Herb^mix-gr^ and Ornip^gr^, particularly on day 28, compared to their values in DexaHepato^gr^. In contrast, although a significant decline in serum activities of liver enzymes at Herb^mix-gr^ or Ornip^gr^, was reported after therapy, on days 21 and 28, serum activities of AST, ALT, and ALP were still significantly higher than their values in Cont^gr^ (day 0). Serum activities of AST, ALT, and ALP were within the reference ranges that were reported by Assawarachan et al. (15), Elhiblu et al. (75), Atata et al. (78), and Kaneko et al. (89). No remarkable changes were noted in serum AST, ALT, and ALP between Herb^mix-gr^ and Ornip^gr^. These findings were consistent with the previously reported findings of Eman et al. (4), Abdou et al. (59), and Khan et al. (91).

Increased serum AST and ALT activities indicated hepatic cell injury with enzyme leakage. A corticosteroid-induced enzyme caused high serum ALP activity, which might be related to glycogen synthesis (87). In animals with hepatocellular injury, increases in either ALT or AST activity demonstrated leakage of the enzymes and hepatocellular membrane damage. An increase in AST serum without increased ALT activity indicated extrahepatic issues, such as muscle injury. Both ALT and AST levels were regarded as hepatocellular “leakage” enzymes. When persistent increases in ALT were characteristic of chronic hepatitis in dogs, this was a sign of liver disease. With an 85% sensitivity, ALP was considered a sensitive marker for cholestasis (4, 15, 83). Many studies referred to the ethanol extract of *C. longa* rhizomes as a promising therapy for preventing and treating liver cirrhosis.

In contrast, a significant reduction in AST, ALT, and ALP serum activities was reported after liver fibrosis treatment with ethanol extract of *C. longa* compared with their serum levels before *C. longa* treatment (30, 33). Pomegranate peel extract had a protective effect against ccl4-induced liver fibrosis and could be used to treat liver fibrosis (40). Pomegranate peel has a protective effect on the liver. It improved the level of liver enzymes such as ALT and AST in liver disease, which were remarkably reduced after being treated with pomegranate peel compared with their levels before pomegranate treatment (70). Furthermore, other articles showed that chronic pomegranate peel extract supplementation reduced oxidative damage to the liver and improved hepatic structure and functions because pomegranate is a popular plant with high nutritive value and high antioxidant activity. It reduced lipid peroxidation and nitric acid in the liver and kidney. Its hepatoprotective effect and highly antioxidant capacity are reflected through its efficacy to significantly lower serum activities of AST, ALT, and ALP in diseased animals with liver fibrosis after treatment with pomegranate (35, 92–94).

Serum concentrations of total bilirubin and direct bilirubin showed no significant changes between Cont^gr^, DexaHepato^gr^, Herb^mix-gr^, and Ornip^gr^. Moreover, they were within the reference ranges mentioned by Elhiblu et al. (75) and Kaneko et al. (89). Serum bilirubin levels might be elevated due to hepatocyte damage and decreased elimination (75). Hepatic abscesses are uncommon in dogs with higher levels of bilirubin, ALP, and ALT (95). Gouda (61) discovered that the total bilirubin level in steroid-induced hepatopathy was remarkably increased by the fourth week compared to a healthy one. Total bilirubin was less sensitive but more specific than liver enzymes in identifying hepatobiliary diseases. Eman et al. (4) mentioned that the bilirubin level at the 14th day of steroid-induced hepatopathy in dogs was within the reference range and did not significantly increase. Small et al. (85) mentioned that serum biochemistry in dogs with gallbladder mucocele revealed increased liver enzymes, including ALP (98.2% of cases), ALT (87.4% of cases), GGT (85.7% of cases), and AST (62.2% of cases). Hyperbilirubinemia is reported in 83.2% of the cases in the current literature (86, 96, 97).

### 4.4 Kidney functions indices

Kidney function indicators displayed remarkable alterations in serum concentrations of BUN and Cr. In contrast, significant elevations in serum levels of BUN and Cr were reported in Cont^gr^ and treated group (Herb^mix-gr^ or Ornip^gr^) compared with their values in DexaHepato^gr^. These essential changes disappeared between Herb^mix-gr^ and Ornip^gr^. Furthermore, they were within their reference ranges reported by Elhiblu et al. (75) and Kaneko et al. (89). Renal dysfunction has been identified as a common complication in patients with advanced liver disease. As a result, the increased BUN and Cr levels could be attributed to impaired kidney function associated with liver cirrhosis due to the liver’s decreased capacity to detoxify harmful products (75). In contrast, Gong et al. (98) stated that the level of Cr decreased after dexamethasone injection, while Assawarachan et al. (15) reported normal blood levels of BUN and Cr in dogs with hepatic disease.

### 4.5 Ultrasonographic findings

In dogs, the liver was composed of four lobes, four sub lobes, and two processes—left lobe (lateral and medial), quadrate lobe, right lobe (lateral and medial), and caudate lobe (caudate and papillary processes) (99, 100), which could not be easily distinguished unless separated by peritoneal effusion. Ultrasonography, liver biopsy, and biochemical analysis complemented each other in diagnosing glucocorticoid-induced hepatopathy and should be used in conjugation to interpret the results (59). The current study revealed Cont^gr^ in 0 days and showed a standard ultrasonographic image of the hepatobiliary system and its vasculatures. In contrast, the hepatic parenchyma appeared uniform and homogenous, with numerous echoes and interruptions caused by the hepatic and portal veins. The echotexture is more hypoechoic compared with the spleen and hyperechoic compared with the kidney. These findings agreed with Penninck and d’Anjou (14), Mannion (52), Rademacher (53), and Ahmed et al. (54). In contrast, the normal liver parenchyma had uniform echogenicity of medium intensity, which was equal to or slightly more echogenic than splenic parenchyma (101). The dimension of the liver could not be effectively established due to the overlapping liver lobes. However, the area where the liver could be imaged had been established at the right abdominal flank region caudal to the ribs (102). The afferent vascular flow to the liver was dual, with a more significant proportion coming from the PV and the rest from the hepatic arteries (99). The efferent flow followed the hepatic veins into the caudal vena cava (CVC). This particular vascular pattern was divided into all hepatic lobes and could aid in distinguishing these lobes (14).

The common suggestions for liver ultrasonographical examination include unexplained weight loss, masses in the liver region, ascites, metastases, fever of unknown origin, abdominal pain, icterus, and trauma (13, 103). Regarding the present results, DexaHepato^gr^ on day 7 in both the herbal mixture and ORNIPURAL® groups showed a slight increase in echogenicity of the hepatic parenchyma. However, it was still less than the spleen reported ultrasonographically. The liver showed a focal hyperechoic area within the liver parenchyma. On day 14, DexaHepato^gr^ showed a diffuse increase in hepatic parenchymal echogenicity and it was hyperechoic compared to the renal cortex and was like echogenicity of splenic parenchyma. The diffuse and homogenous hyperechogenicity of the hepatic parenchyma in hepatopathy-infected dogs resulted in a significant decrease in clearance of the hepatic vessel wall. These results were in agreement with Rademacher (53), Ahmed et al. (54), Abdou et al. (59), Gouda (61), and Nyland et al. (103). The hepatic parenchyma sometimes appeared heterogeneous and contained hypoechoic foci, most likely due to concurrent nodular hyperplasia. Vacuolar hepatopathy associated with steroid hepatopathy and hepatic lipidosis was common in small animals and often occurred with other primary disorders (53). These findings were supported by Abdou et al. (59), who observed a slight increase in hepatic echogenicity compared with the liver-spleen echogenicity on day 7 in dogs with experimentally induced steroid hepatopathy, which reflected the hepatic damage due to vacuolation of hepatocytes (104). These findings were supported by cytological and histopathologic findings that revealed mild to moderate hepatic vacuolation. At days 14, 21, and 28, a pronounced and progressive increase in hepatic echogenicity was noticed, which reflected the advanced hepatic damage due to the vacuolation of hepatocytes (59, 104). Moreover, Gouda (61) reported that the glucocorticoid-induced hepatopathy allowed glycogen accumulation in the hepatic cells, which appeared as granules caused hepatocellular granulation, so parenchymal echogenicity was increased gradually by prolonging the duration of using of glucocorticoids, as begin as focal changes then become diffuse. In steroid-induced hepatopathy, the liver parenchyma appears more hypoechoic than the spleen. It became isoechoic in the first week of the experiment, and the liver became more hyperechogenic than the spleen.

On the other hand, unfortunately, ultrasound was good at identifying lesions but not so good at differentiating lesion type (13). Moreover, Eman et al. (4) reported a diffuse decrease in hepatic echogenicity in acute hepatitis on ultrasonography while an increase in hepatic echogenicity and anechoic ascetic fluid in chronic hepatitis. Sen et al. (12) described the diagnosis of ascites of hepato-cardiac origin in a 2 years old Labrador Retriever bitch using ultrasonography in which the animal was suffering from chronic cough and recurrent ascites for more than 6 months. The physical examination revealed ascites, confirmed through ultrasonography and hepatic cyst. However, the whole blood picture indices and serum biochemical parameters were standard.

After 7 days of treatment with an herbal mixture of *C. longa* extract and pomegranate peel extract, hepatic ultrasonography of examined dogs shoed that liver parenchyma returned to normal, and the hepatic parenchyma appeared as uniform homogenous numerous echoes. The same ultrasonographic findings were reported for the liver at Herb^mix-gr^ (Day 28). In contrast, after 7 days of treatment with ORNIPURAL^®^, the diffuse and homogenous hyperechogenicity of the hepatic parenchyma was still visualized in hepatopathy-infected dogs, resulting in a significant decrease in clearance of the portal vein wall and more clearance to the hepatic vein. After 14 days of treatment with ORNIPURAL^®^, hepatic ultrasonography of Ornip^gr^ on day 28 showed that liver parenchyma became slightly normal as it had uniform homogenous numerous echoes; however, the liver had an increase in its parenchymal echogenicity. Ahmed et al. (54) reported that after 7 days of treatment with *C. longa* extract in dogs with steroidal-induced hepatopathy, the ultrasonographical picture of the liver showed a slight decrease in echogenicity of the liver parenchyma, which began to improve on day 14 of treatment. Liver parenchyma improved more nearly to become normal. Furthermore, Kozat and Sepehrizadeh (83) added that hepatic lipidosis could be diagnosed slightly more accurately than other diffuse hepatic diseases. Commonly, ultrasonography has been confirmed to be an effective tool for evaluating the progression of canine hepatopathy that might be owning to its ability to assess the hepatic parenchymal structure (105) and subjective visual comparison of liver echogenicity with normal spleen was an accepted method for detecting diffuse changes in hepatic echogenicity (59, 82, 106).

### 4.6 Histopathological findings

Histopathology was still regarded as the diagnostic gold standard (107). A liver biopsy was necessary for the diagnosis of canine liver disease. A biopsy must reliably represent the abnormalities throughout the hepatic parenchyma to diagnose diffuse liver disease accurately. Liver biopsy should be collected from more than one lobe (107, 108). The current study mentioned that histopathological findings of the liver in control healthy dogs (Day 0) showed normal parenchyma as the hepatic parenchyma contains plates and sinusoids, which were usually one cell thick and have a regular arrangement around the central hepatic vein, causing a radiating pattern of the sinusoidal network. Individual hepatocytes were polygonal, had a pale to eosinophilic cytoplasm, clearly outlined cell margins, the nucleus was centrally placed, and one or more nucleoli might be recognized. These findings matched those of Ahmed et al. (54), Hübscher (107), and Cullen et al. (109).

Abdou et al. (59) confirmed that the ultrasonographic findings were usually supported by cytological and histopathologic findings in dogs with glucocorticoid-induced hepatopathy, which revealed that nearly all the hepatocytes were distended by multiple vacuoles of different sizes with little visible cytoplasm, where the hepatocytes appeared as large vacuolated cells with a peripherally positioned nucleus. These hepatic morphologic alterations have been attributed variably to intercellular edema (110), accumulation of cytoplasmic lipids (111), or glycogen (112). The current study showed that DexaHepato^gr^ on day 14 in each herbal mixture group and ORNIPURAL^®^ group showed histopathological changes in the liver of dogs with steroidal-induced hepatopathy. In contrast, hydropic degenerations were visualized within hepatic parenchyma (55%) and thrombus formation within the hepatic blood vessels adjacent to necrotic tunica intima. These findings were consistent with Eman et al. (4), Ahmed et al. (54), Abdou et al. (59), French et al. (113), and Pereira et al. (114). Exogenous or endogenous glucocorticoids excess was associated with glycogen accumulation in the cytoplasm of hepatocytes, which appeared as distended to two to three times the size of normal hepatocytes with lacey and wispy vacuolation on histopathological examination (113). Swollen hepatocytes with eosinophilic cytoplasm strands without nucleus displacement from the center were the significant histopathological changes in steroid-induced hepatopathy in dogs (109, 115).

After 14 days of treatment with an herbal mixture of *Curcuma longa* extract and pomegranate peel extract, Herb^mix-gr^ reported histopathological changes in the liver sections. In contrast, they showed that majority of the hepatic parenchyma appeared normal. However, the focal necrotic area infiltrated with mononuclear cells associated with microsteatosis in some hepatocytes (15%) was visualized. Dilated hepatic blood vessels and dilated sinusoids were also encountered. These results agreed with Salama et al. (30) and Farjam et al. (33). The current study also added that the histopathological findings of hepatic parenchyma in steroidal-induced hepatopathy dogs after 14 days of treatment with ORNIPURAL® at Ornip^gr^ (day 28) showed normal hepatic parenchyma. However, unicellular degenerative (10%), necrotic changes of some hepatocytes, and congestion of some portal veins were also noticed. Ahmed et al. (54) reported that after 7 days of treatment with *C. longa* extract in dogs with steroidal-induced hepatopathy, the histopathological changes in the liver sections showed randomly distributed degenerative changes, mainly steatosis (25%) and acute cell swelling beside the presence of hemosiderin-laden macrophages and round cells infiltration around the central veins. Badylak and Van Vleet (116) stated that corticosteroids caused a characteristic pattern of vascularization, cytoplasmic vacuolation, and glycogen accumulation within hepatocytes when given to dogs. Eman et al. (4) mentioned that cytology revealed hepatic vacuolar degeneration, and histopathology revealed necrosis and apoptosis of hepatocytes in acute hepatitis while revealing massive fibrous tissue proliferation in hepatic parenchyma in chronic hepatitis.

## 5 Conclusion

The present study reported the most remarkable efficacy of both ORNIPURAL^®^ and herbal mixture of *C. longa* extract and pomegranate peel extract as hepatoprotective medicaments in dogs’ therapy of dexamethasone-induced fatty liver. Therefore, 14 days of treatment with either a herbal mixture or ORNIPURAL^®^ in treated dogs (treatment groups) induced an unmistakable improvement in their clinical status, blood pictures, and serum hepatorenal parameters as well as characteristic sonographic and histopathological findings compared with those in dexamethasone-induced hepatic lipidosis (hepatopathy groups). Compared to dogs treated with ORNIPURAL^®^, this clinical improvement was more evident in dogs treated with an herbal mixture. Consequently, all abnormal clinical findings disappeared in dogs treated with an herbal mixture on day 14 following therapy. However, these findings were still observed to a lesser degree in dogs treated with ORNIPURAL^®^. Moreover, no significant alterations in blood pictures and serum hepatorenal indices were demonstrated between ORNIPURAL^®^ and herbal-treated dogs. Overall, the herbal mixture of *C. longa* extract and pomegranate peel extract had higher efficacy and greater potency than conventional therapy that uses ORNIPURAL® in treating dogs with hepatopathy. Moreover, the study also recommended the parallel use of this herbal mixture as well as ORNIPURAL® in long-term therapeutic strategies in dogs with dexamethasone-induced fatty liver as both subsided and minimized dexamethasone side effects. After 14 days of treatment with either an herbal mixture or with ORNIPURAL^®^, the histopathological changes in the liver sections of treated dogs showed that majority of the hepatic parenchyma appeared normal. Ultrasonography, as the sole diagnostic technique, was not enough to evaluate hepatobiliary disorders in canines. Thus, there was a need for other diagnostic tools such as clinical findings, laboratory assays, histopathology, and others.

## Data Availability

The raw data supporting the conclusions of this article will be made available by the authors, without undue reservation.
