# Supplementary material for: ORNIPURAL® as conventional therapy versus mixture of Curcuma longa extract and pomegranate peel extract as homeotherapy in dogs with dexamethasone-induced hepatopathy: clinicolaboratory, ultrasonographic, and histopathological monitoring
Source: Front Vet Sci. 2025 Apr 9;12:1564648. doi: 10.3389/fvets.2025.1564648 (PMC12016885; doi:10.3389/fvets.2025.1564648)
Supplement: Supplementary file 1 [file Table_1.docx]

**Supplementary Table 1.** Methodology plan of the experimental study in investigated dogs.

|  | **Clinical examination** | | | | | **whole blood picture** | | | | | **Serum B.C.** | | | | | **U.E.** | | | | | **Histopathology** | | | | |
| --- | --- | --- | --- | --- | --- | --- | --- | --- | --- | --- | --- | --- | --- | --- | --- | --- | --- | --- | --- | --- | --- | --- | --- | --- | --- |
|  | Day 0^#^ | Day 7^*^ | Day 14^*^ | Day 21^¶^ | Day 28^¶^ | Day 0^#^ | Day 7^*^ | Day 14^*^ | Day 21^¶^ | Day 28^¶^ | Day 0^#^ | Day 7^*^ | Day 14^*^ | Day 21^¶^ | Day 28^¶^ | Day 0^#^ | Day 7^*^ | Day 14^*^ | Day 21^¶^ | Day 28^¶^ | Day 0^#^ | Day 7^*^ | Day 14^*^ | Day 21^¶^ | Day 28^¶^ |
| **Cont^gr^**  **(n=30)** | √ | × | × | × | × | √ | × | × | × | × | √ | × | × | × | × | √ | × | × | × | × | √ | × | × | × | × |
| **D_exa_Hepato^gr^ (n=29)** | × | √ | √ | × | × | × | √ | √ | × | × | × | √ | √ | × | × | × | √ | √ | × | × | × | × | √ | × | × |
| **Herb^mix-gr^**  **(n=14)** | × | × | × | √ | √ | × | × | × | √ | √ | × | × | × | √ | √ | × | × | × | √ | √ | × | × | × | × | √ |
| **Ornip^gr^**  **(n=14)** | × | × | × | √ | √ | × | × | × | √ | √ | × | × | × | √ | √ | × | × | × | √ | √ | × | × | × | × | √ |

Cont^gr^: Control group. D_exa_Hepato^gr^: Steroidal-induced hepatopathy group. Herb^mix-gr^: Herbal mixture treated group. Ornip^gr^: ORNIPURAL® treated group. Serum B.C.: Serum biochemical assays (liver and renal functions bio-markers). U.E.: Ultrasonographic examination. ^#^Control. ^*^Dexamethasone treatment. ^¶^Herbal mixture or ORNIPURAL® treatment. √: conducted. ×: Not conducted.

**Supplementary Table 2.** The most common clinical findings in investigated dogs.

|  | **Gait act** | | | | | **Intake** | | | | | **Emaciation** | | | | | **Sk-Rs** | | | | | | **Skin abscess** | | | | | **R. sk. abscess** | | | | | **Skin lac.** | | | | | | **Lethargy** | | | | | **Dehydration** | | | | | |
| --- | --- | --- | --- | --- | --- | --- | --- | --- | --- | --- | --- | --- | --- | --- | --- | --- | --- | --- | --- | --- | --- | --- | --- | --- | --- | --- | --- | --- | --- | --- | --- | --- | --- | --- | --- | --- | --- | --- | --- | --- | --- | --- | --- | --- | --- | --- | --- | --- |
| **Days** | 0^#^ | 7^*^ | 14^*^ | 21^¶^ | 28^¶^ | 0^#^ | 7^*^ | 14^*^ | 21^¶^ | 28^¶^ | 0^#^ | 7^*^ | 14^*^ | 21^¶^ | 28^¶^ | | 0^#^ | 7^*^ | 14^*^ | 21^¶^ | 28^¶^ | 0^#^ | 7^*^ | 14^*^ | 21^¶^ | 28^¶^ | 0^#^ | 7^*^ | 14^*^ | 21^¶^ | 28^¶^ | 0^#^ | 7^*^ | 14^*^ | 21^¶^ | 28^¶^ | 0^#^ | | 7^*^ | 14^*^ | 21^¶^ | 28^¶^ | 0^#^ | 7^*^ | 14^*^ | 21^¶^ | 28^¶^ |  |
| **Cont^gr^** | ++ |  |  |  |  | ++ |  |  |  |  | XX |  |  |  |  | | XX |  |  |  |  | XX |  |  |  |  | XX |  |  |  |  | XX |  |  |  |  | XX | |  |  |  |  | XX |  |  |  |  |  |
| **D_exa_Hepato^gr^** |  | - | -- |  |  |  | - | -- |  |  |  | √ | √√ |  |  | |  | √ | √√ |  |  |  | √ | √√ |  |  |  | XX | √√ |  |  |  | XX | √√ |  |  |  | | √ | √√ |  |  |  | √ | √√ |  |  |  |
| **Herb^mix-gr^** |  |  |  | ++ | ++ |  |  |  | ++ | ++ |  |  |  | XX | XX | |  |  |  | XX | XX |  |  |  | XX | XX |  |  |  | XX | XX |  |  |  | XX | XX |  | |  |  | XX | XX |  |  |  | XX | XX |  |
| **Ornip^gr^** |  |  |  | + | + |  |  |  | + | + |  |  |  | XX | X | |  |  |  | X | XX |  |  |  | X | XX |  |  |  | X | XX |  |  |  | X | XX |  | |  |  | X | X |  |  |  | X | X |  |

Gait act: Gait and activity (Alert and active). Intake: Feed & water intake. Sk-Rs: Skin rashes and alopecia. R. Sk. Abscess: Ruptured skin. Lacr.: Lacerations. Cont^gr^: Control group. D_exa_Hepato^gr^: Steroidal-induced hepatopathy group. Herb^mix-gr^: Herbal mixture treated group. Ornip^gr^: ORNIPURAL® treated group. ^##^Control. ^*^Dexamethasone treatment. ^¶^Herbal mixture or ORNIPURAL® treatment. √: Observed. √√: Clearly observed. X: Not completely disappeared. XX: Completely disappeared or not observed. ++: Normal. +: Relatively normal. -: Slightly reduced --: Severely reduced.
